# The Functional Interplay between Protein Kinase CK2 and CCA1 Transcriptional Activity Is Essential for Clock Temperature Compensation in Arabidopsis

**DOI:** 10.1371/journal.pgen.1001201

**Published:** 2010-11-04

**Authors:** Sergi Portolés, Paloma Más

**Affiliations:** Consortium CSIC-IRTA-UAB, Centre for Research in Agricultural Genomics (CRAG), Department of Plant Molecular Genetics, Barcelona, Spain; The University of North Carolina at Chapel Hill, United States of America

## Abstract

Circadian rhythms are daily biological oscillations driven by an endogenous mechanism known as circadian clock. The protein kinase CK2 is one of the few clock components that is evolutionary conserved among different taxonomic groups. CK2 regulates the stability and nuclear localization of essential clock proteins in mammals, fungi, and insects. Two CK2 regulatory subunits, CKB3 and CKB4, have been also linked with the *Arabidopsis thaliana* circadian system. However, the biological relevance and the precise mechanisms of CK2 function within the plant clockwork are not known. By using ChIP and Double–ChIP experiments together with *in vivo* luminescence assays at different temperatures, we were able to identify a temperature-dependent function for CK2 modulating circadian period length. Our study uncovers a previously unpredicted mechanism for CK2 antagonizing the key clock regulator CIRCADIAN CLOCK-ASSOCIATED 1 (CCA1). CK2 activity does not alter protein accumulation or subcellular localization but interferes with CCA1 binding affinity to the promoters of the oscillator genes. High temperatures enhance the CCA1 binding activity, which is precisely counterbalanced by the CK2 opposing function. Altering this balance by over-expression, mutation, or pharmacological inhibition affects the temperature compensation profile, providing a mechanism by which plants regulate circadian period at changing temperatures. Therefore, our study establishes a new model demonstrating that two opposing and temperature-dependent activities (CCA1-CK2) are essential for clock temperature compensation in Arabidopsis.

## Author Summary

Most organisms are able to rhythmically coordinate their physiology and metabolism in consonance with the day-night cycle. The cellular mechanism responsible for generating the biological rhythms is known as circadian clock. In contrast to many other biochemical reactions, the pace of the clock does not change with temperature (i.e. the clock does not run faster at high temperatures). This is a property known as clock temperature compensation, and despite its importance for circadian function, we know few details about the mechanisms responsible for the temperature-dependent regulation of circadian period length. Plants, as sessile organisms, are particularly sensible to maintaining the clock running at the proper pace, regardless the external temperature. In our study, we have identified that the activity of two key plant clock components (CK2 and CCA1) is regulated by temperature. Notably, these temperature-dependent activities are antagonistic, and they counterbalance each other at increasing temperatures so that the clock does not run faster at these high temperatures. When we alter the expression and activity of CK2 and CCA1, we observe that the clock is not longer able to maintain the proper pace, thus demonstrating that the balance between these two activities is essential for clock temperature compensation in plants.

## Introduction

Circadian rhythms are daily biological oscillations driven by an endogenous mechanism known as circadian clock. The phase of the rhythms is synchronized by environmental cues, mostly changes in light and temperature, that occur during the 24-hour day/night cycle. Synchronization ensures adequate timing and allows the rhythmic activities to occur at the most appropriate phase relationships with the environment [1]–[5]. In many organisms, the reciprocal regulation among key clock genes and proteins sustains molecular oscillations that are translated into metabolic and behavioral rhythms [6]–[8]. Additional mechanisms involving chromatin remodeling [9], [10] and post-translational regulation of clock components [11], [12] also contribute to circadian rhythmicity. Despite the conservation of clock mechanisms, the actual molecular components responsible for circadian function are not conserved among phylogenetic kingdoms. A remarkable exception is the protein kinase CK2 (formerly casein kinase 2) with an important function within the plant, fungi, insect and mammalian circadian systems [13].

CK2 is an evolutionarily conserved serine/threonine protein kinase involved in the regulation of key cellular events including tumorigenesis, cell viability and proliferation [14]. CK2 achieves its function by regulating more than 300 putative substrates [15]. The CK2 holoenzyme consists of two catalytic α-subunits and two regulatory β-subunits forming a hetero-tetrameric (α2β2) structure [16]. On a broad sense, the regulatory CK2β subunits provide the substrate selectivity and increase the overall catalytic activity [17], [18]. Regarding the circadian function, CK2 has emerged as a conserved molecular component modulating the subcellular localization and stability of key clock proteins [13]. For example, CK2 regulates the nuclear localization of the mammalian clock component BMAL1 [19] and the protein stability of PERIOD2 (PER2) [20], [21]. These findings are consistent with studies in *Drosophila melanogaster* [22], [23] showing that CK2 regulates the subcellular distribution and stability of the core components PERIOD (PER) and TIMELESS (TIM) [22]–[24]. In plants, two of the four members of the Arabidopsis family of CK2 regulatory subunits, CKB3 [25]–[27] and CKB4 [28], [29] have been also functionally linked with the plant circadian clock. CK2 phosphorylates the Arabidopsis central clock components CIRCADIAN CLOCK-ASSOCIATED 1 (CCA1) and LATE ELONGATED HYPOCOTYL (LHY) [26]. Furthermore, the CCA1 phosphorylation was proposed to be important for CCA1 clock function [25]. Over-expression of CKB3 or CKB4 leads to period shortening [27], [28] and altered day-length-dependent regulation of developmental outputs [27], [28]. The pervasive alterations of many clock outputs and the changes in oscillator expression suggested that CK2 might be closely regulating the oscillator function.

Despite all these advances, little is known about the actual mechanism of CK2 function within the Arabidopsis circadian clock. In contrast, a recent study on the *Neurospora crassa* circadian system has importantly advanced our knowledge of CK2 role controlling a defining property of circadian function [30]. Indeed, to be effective as a timing mechanism, the circadian system must be relatively independent of temperature changes in order to avoid running faster at higher temperatures and slower at lower temperatures [31]. This property was proposed to rely on a compensating mechanism, which would allow the clock to buffer its period length against changes in temperature [32]. The recent study by Mehra et al., assigns a role for CK2 in the mechanism underlying temperature compensation in Neurospora. The mechanism seems to involve CK2-mediated phosphorylation at specific sites of the period-controlling clock protein FREQUENCY (FRQ). The CK2-mediated phosphorylation of FRQ targets the protein for degradation preferentially at high temperatures, thereby contributing to temperature compensation in Neurospora [30].

Experimental data and computer modeling studies have suggested different mechanisms contributing to temperature compensation. One of the mechanisms include the counterbalance of opposing biochemical functions that have similar temperature coefficients [33]. Consistently, the contrasting effects of inter- and intra-molecular interactions of PER were proposed as a basic mechanism underlying temperature compensation in Drosophila [34]. The key role of Drosophila PER was further reinforced by natural variation studies showing that *per* polymorphisms might help to fine-tune the circadian clock to different thermal environments [35]. A more recent study has also shown that the interaction between the Drosophila circadian photoreceptor CRYPTOCHROME (CRY) and the protein complex composed of PER and TIM are also critical for temperature compensation in Drosophila [36]. In other model organisms, the mechanisms by which the circadian clock compensates its period length over a range of temperatures are less well known. In the cyanobacterium *Synechococcus elongatus*, the ATPase activity of the essential clock component KaiC was found to be temperature compensated. The authors suggested that temperature compensation in Synechococcus could be driven by this biochemical reaction [37]. In plants, the temperature compensation of leaf movement rhythms was uncovered by a quantitative genetic approach [38]. The identified genes included the flowering time regulator *FLOWERING LOCUS C* [39] and the flowering and clock-related gene *GIGANTEA* (*GI*) [40]. GI was proposed to play a role extending the temperature range over which rhythms can be maintained [40]. By using mutant plants and analyzing the expression of the central clock genes *CCA1* [41], and *LHY* [42] and *TIMING OF CAB EXPRESSION 1* (*TOC1* or *PRR1*) [43], [44], the authors also concluded that a balance between GI and the core component LHY was important at high temperatures, while CCA1 would preferentially function at low temperatures [40]. Although quantitative genetic studies have been a successful approach for the identification of possible elements contributing to temperature compensation, the mechanisms responsible for these clock responses remain to be discovered.

Here, we provide evidence for a role of CK2 modulating temperature responses in Arabidopsis. This function is achieved by antagonizing the clock component CCA1. CK2 does not alter protein accumulation or subcellular localization, as CK2 does in other circadian systems, but affects the transcriptional activity of CCA1. Over-expression, mutation or pharmacological inhibition reveal that both CK2 regulatory function and CCA1 binding are more effective at high temperatures, which provide a mechanism by which plants buffer clock period length against temperature changes.

## Results

### Genetic interaction of CCA1 and CKB4 in the control of flowering time, hypocotyl elongation, and circadian gene expression

**Figure 1:**
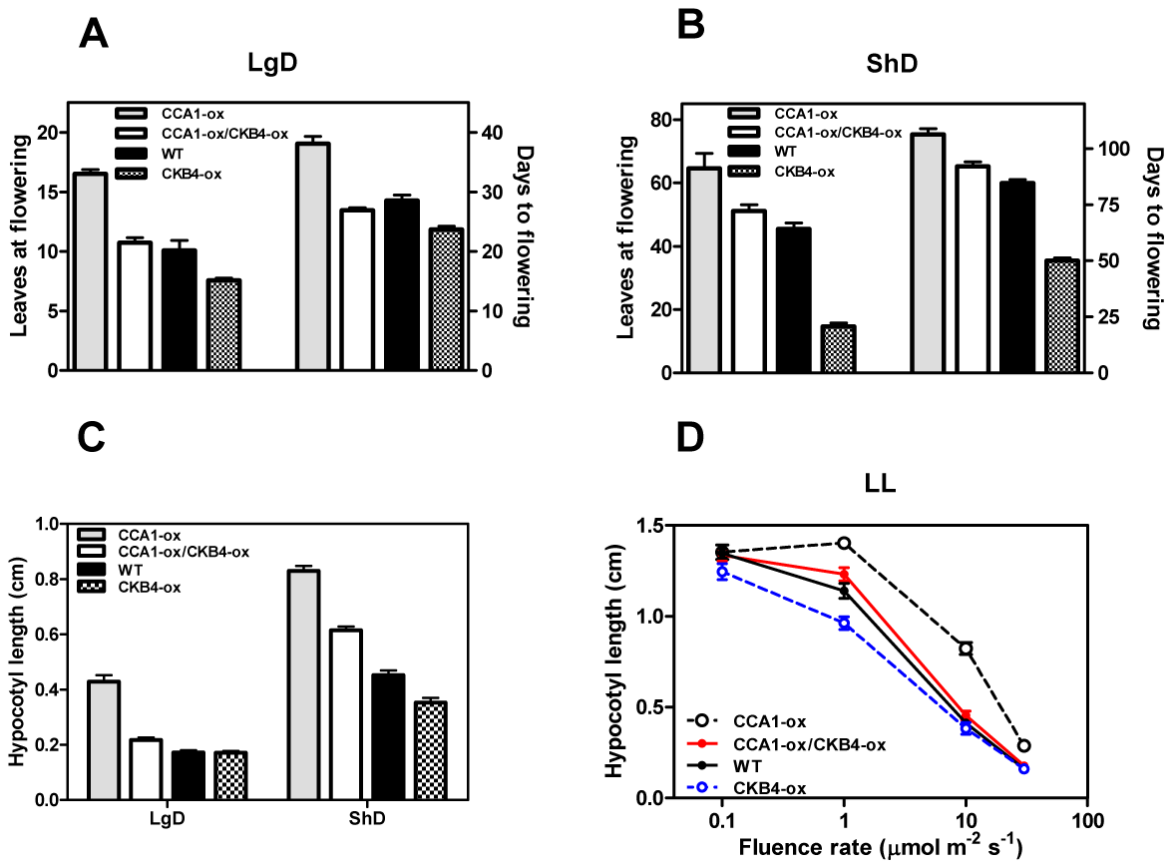
Genetic interaction of CCA1 and CKB4. Flowering time of plants grown under LgD (16 h light:8 h dark) (A) or ShD (8 h light:16 h dark) (B). The number of leaves at flowering and the number of days to flowering are presented. Data is shown as means ± SEM of three independent experiments. (C) Hypocotyl lengths of seedlings grown under LgD or ShD. Data is shown as means ± SEM of 15–20 seedlings. (D) Hypocotyl lengths of seedlings under different intensities of white light (LL). Data is shown as means ± SEM of 15–20 seedlings. All the experiments were performed at least twice with similar results to those shown here.

The circadian clock is responsible for the integration of temporal and photic information that regulates hypocotyl elongation [45], [46] and flowering time in Arabidopsis [47]–[49]. Previous studies have shown similar circadian phenotypes of *cca1* mutant and CKB4 over-expressing (CKB4-MYC-ox) plants [29]. We therefore explored the inverse correlation between phenotypes and expression by conducting a genetic study in which CCA1 over-expressing (CCA1-YFP-ox) and *cca1-1/lhyRNAi* mutant plants [50] were transformed with the CKB4-MYC-ox construct [29]. We used homozygous, single insertion lines (Figure S1) to examine hypocotyl elongation and flowering time as an indication of clock function on these developmental outputs. Analysis of CCA1-YFP-ox/CKB4-MYC-ox plants revealed that over-expression of CKB4 reduced the delayed flowering phenotype of CCA1-YFP-ox plants such that flowering in CCA1-YFP-ox/CKB4-MYC-ox plants occurred at almost the same time than in Wild-Type (WT) plants under both Long-Days (LgD,16 h light:8 h dark) (Figure 1A) or Short-Days (ShD, 8 h light:16 h dark) (Figure 1B). In contrast, the early flowering phenotype of *cca1-1/lhyRNAi* mutant plants was not significantly affected by over-expression of CKB4 (Figure S2), which indicates a non-additive interaction and suggests that the effect of CKB4-MYC-ox might require the functional expression of *CCA1* and *LHY*. When hypocotyl elongation was examined under light:dark (LD) cycles, we found that over-expression of CKB4 significantly reduced the long hypocotyl phenotype of CCA1-YFP-ox plants under both LgD (p-value<0.0001) or ShD (p-value<0.0001)(Figure 1C). A similar trend was observed at different fluence rates of constant white light (Figure 1D). Together, these results indicate that the severity of the flowering and hypocotyl phenotypes of CCA1-YFP-ox plants is considerably reduced by over-expression of CKB4.

As CCA1 regulates the expression of the evening-expressed clock gene *TOC1* [51], [52], we next explored whether over-expression of CKB4 altered the repressive function of CCA1. Luminescence from WT, CCA1-YFP-ox, CKB4-MYC-ox and CCA1-YFP-ox/CKB4-MYC-ox plants expressing the *TOC1* promoter fused to the luciferase (*TOC1:LUC*) was examined under 12 h light/12 h dark (LD) conditions. As expected, *TOC1:LUC* expression in CCA1-YFP-ox plants exhibited a very reduced amplitude compared to WT plants (Figure S3). In contrast, higher amplitude and slightly advanced phase of *TOC1* promoter activity was observed in CKB4-MYC-ox plants (Figure S3). Noticeably, the double over-expressing CKB4 and CCA1 plants displayed circadian waveforms very similar to those observed in WT plants (Figure S3). Consistent with the studies of hypocotyl length and flowering time, these results suggest that the *TOC1:LUC* repression by CCA1-YFP-ox is reduced by over-expression of CKB4. These results are also in agreement with the notion that CKB4 interferes with CCA1 function within the circadian clock.

### Molecular interaction of CCA1 and CKB4 in the nucleus

**Figure 2:**
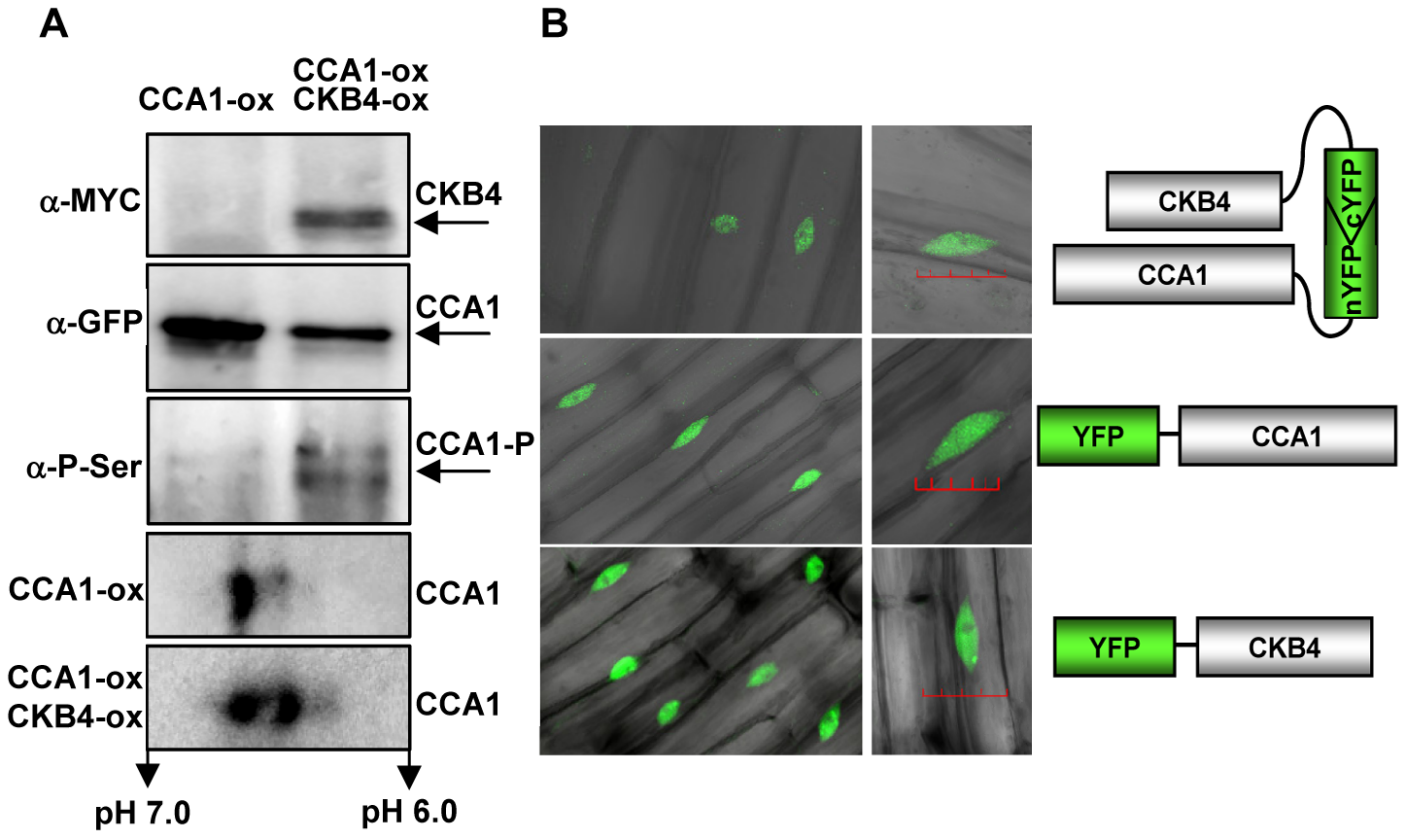
Molecular interaction of CCA1 and CKB4. (A) Western-blot analysis of protein extracts immunoprecipitated with anti-GFP antibody (α-GFP) and subsequent detection of CKB4-MYC (α-MYC), CCA1-YFP (α-GFP) or phosphorylated CCA1 isoforms (CCA1-P)(α-PSer). Lower panels show the immunoblot analysis of two-dimensional gels detecting CCA1-YFP with α-GFP. In all cases, plants were grown under LD conditions and samples were collected at ZT3. All the experiments were performed at least twice with similar results to those shown here. (B) Confocal microscopy analysis of seedlings over-expressing CCA1 fused to the N-terminal fragment of YFP and CKB4 fused to the C-terminal fragment (top panel). Images of CCA1 (middle panel) and CKB4 (lower panel) fused to full-length YFP are also shown. Scale bar 0.2 µm.

Previous studies have shown the interaction of CCA1 with CKB1, CKB2 and CKB3, three members of the CK2 regulatory subunit family with high sequence homology to CKB4 [26]. To examine the possible physical association of CCA1 with CKB4, we performed *in vivo* co-immunoprecipitation assays using the CCA1-YFP-ox/CKB4-MYC-ox plants. Immunoprecipitation of CCA1 with the anti-GFP antibody (α-GFP) and subsequent detection with the anti-MYC antibody (α-MYC) revealed a band with a relative molecular mass of about 40 kDa, coincident with the expected size of CKB4-MYC (Figure 2A, α-MYC) indicating that CCA1 and CKB4 can be found in the same protein complex in Arabidopsis plants. The absence of signal in single CCA1-YFP-ox extracts (Figure 2A, α-MYC) and single CKB4-MYC-ox (not shown) revealed the specificity of the interaction. Detection using the GFP antibody showed a protein band with a molecular mass of 110 kDa that coincided with the predicted size of the CCA1:YFP fusion protein and confirmed the immunoprecipitation of CCA1 (Figure 2A, α-GFP). The interaction was also examined in plants expressing CCA1 under its own promoter (CCA1pro:CCA1-MYC/*cca1-1*) [53]. The results confirmed that CCA1 and CK2 regulatory subunits are present in the same protein complex (Figure S4) as revealed after immunoprecipitation with an antibody to the human regulatory subunit CK2B (α-CKB) followed by detection with the α-MYC antibody. The α-CKB antibody efficiently recognizes the Arabidopsis CK2 regulatory subunits (Figure S4). In agreement with previous studies showing that CK2 phosphorylates CCA1 [26], our immunoprecipitation assays with anti-GFP and subsequent detection with an antibody that specifically recognizes phosphorylated serine residues (α-PSer) revealed that the pattern of CCA1 phosphorylation was increased in CCA1-YFP-ox/CKB4-MYC-ox plants as compared with single CCA1-YFP-ox plants (Figure 2A, α-PSer). The data was reinforced by two-dimensional protein gel analysis followed by immunoblotting with the GFP antibody (Figure 2A, lower panels). The results showed that over-expression of CKB4 enriched CCA1 spots with a lower isoelectric point, which most likely correspond to CCA1 phosphorylated isoforms. Together, these results are consistent with previous published studies and suggest that CK2 phosphorylates CCA1 most likely by direct interaction with the CK2 regulatory subunits.

We next examined the *in vivo* subcellular localization of CCA1 and CKB4 interaction by exploiting Bimolecular Fluorescent Complementation (BiFC) assays [54]. We analyzed plants over-expressing both CKB4 fused to the C-terminal fragment of the Yellow Fluorescent Protein (YFP) and CCA1 fused to the YFP N-terminal fragment. Confocal microscopy analysis revealed fluorescent signals accumulating in the nucleus (Figure 2B, CCA1-nYFP-ox, CKB4-cYFP-ox). As fluorescence would be only visualized if the two fragments of YFP are in such a close proximity that the fluorophore is reconstituted, our results reflect the *in vivo* interaction of CCA1 and CKB4 in the nucleus. The subcellular localization of CCA1 and CKB4 interaction is similar to the localization observed in plants expressing the proteins fused to the full-length YFP. In both cases, the localization was mostly nuclear, in speckles and homogeneous distribution throughout the nucleoplasm for CKB4 (Figure 2B, CKB4-YFP) and mostly in nuclear speckles for CCA1 (Figure 2B, CCA1-YFP). Similar fluorescent signals were observed in CCA1-ox plants over-expressing CKB4 fused to full-length YFP or in CKB4-MYC-ox plants over-expressing CCA1 fused to full-length YFP (Figure S4). In contrast, no evident fluorescence could be detected when BiFC experiments were performed with plants over-expressing both TOC1 (TOC1-nYFP-ox) and CKB4-cYFP-ox (Figure S4).

### CK2 activity antagonizes CCA1 regulatory function without affecting CCA1 protein accumulation

**Figure 3:**
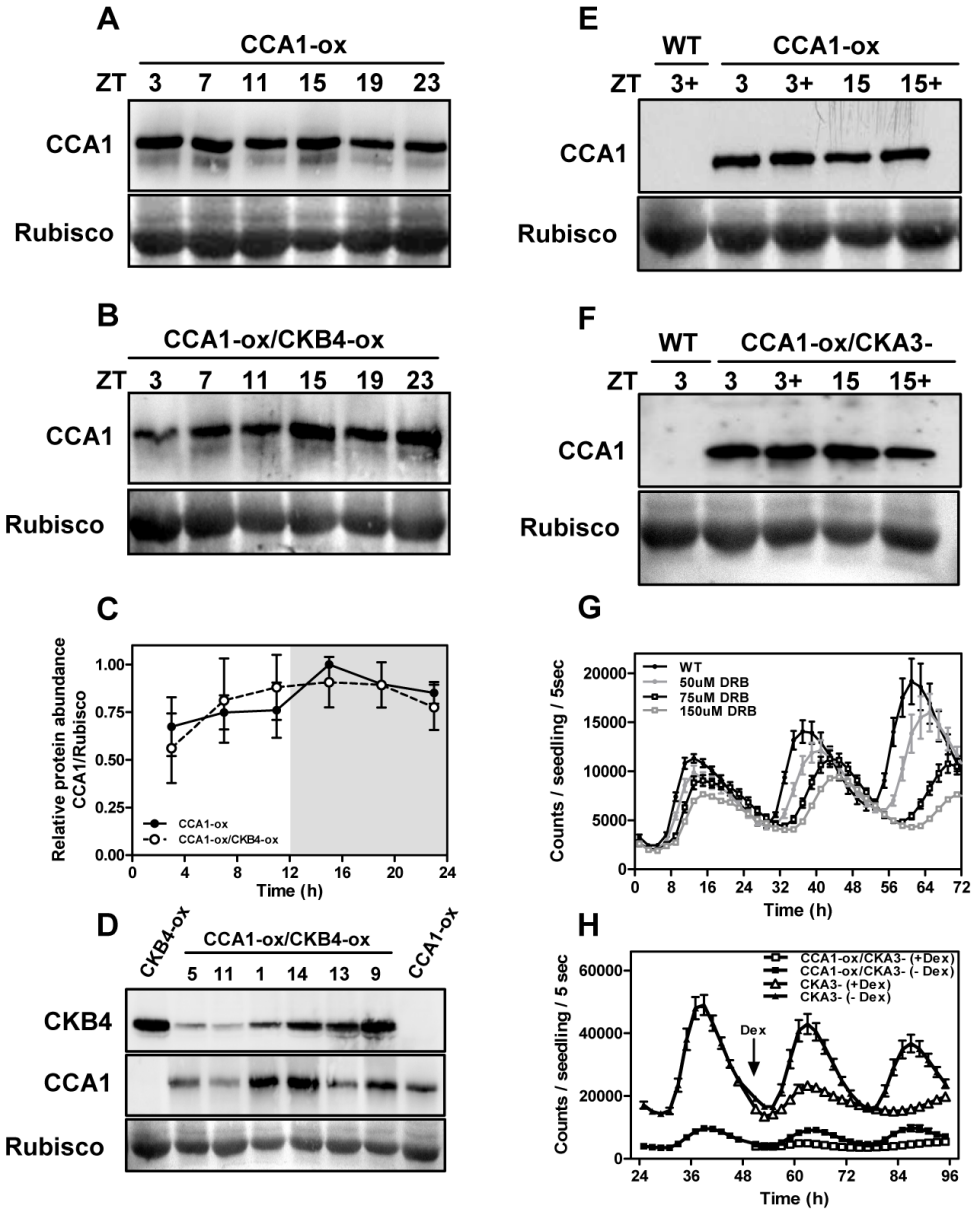
CK2 regulates *TOC1:LUC* expression without altering CCA1 protein accumulation. Immunodetection of CCA1 protein with α-GFP in CCA1-YFP-ox (A) or CCA1-YFP-ox/CKB4-MYC-ox plants (B). Seedlings were entrained under LD cycles and samples were collected at the indicated ZT. Quantification of CCA1-YFP protein accumulation is shown in (C). Means ± SD of two independent experiments are represented relative to the maximum value and normalized to the RUBISCO protein. (D) Western-blot analysis of CKB4-MYC (α-MYC) or CCA1-YFP (α-GFP) in CCA1-YFP-ox, CKB4-MYC-ox and different CCA1-YFP-ox/CKB4-MYC-ox lines. Western-blot analysis of CCA1-YFP protein accumulation in CCA1-YFP-ox plants in the presence (+) or absence of 150 µM DRB (E) or in CCA1-YFP-ox/CKA3- plants with (+) or without 1 µM Dex (F). Samples were collected at ZT3 and ZT15. (G) *TOC1:LUC* luminescence in WT plants treated with DRB (0, 50, 75, 150 µM) or (H) in CKA3- and CCA1-YFP-ox/CKA3- plants in the absence or in the presence of 1 µM Dex added at CT2 (arrow). Seedlings were entrained under LD cycles and transferred to LL prior luminescence recording. Data are represented as means ± SEM of luminescence signals from at least 12 independent plants. All the experiments were performed at least twice with similar results to those shown here.

As phosphorylation targets many clock proteins for degradation [55], we compared by Western-blot analysis, CCA1 protein abundance in CCA1-YFP-ox and CCA1-YFP-ox/CKB4-MYC-ox plants over a diurnal cycle. Our results showed a very similar pattern of CCA1 accumulation in both genotypes at every time point examined (Figure 3A–3C). Furthermore, analysis of CKB4 and CCA1 protein abundance in different CCA1-YFP-ox lines transformed with the CKB4-MYC-ox construct showed a lack of correlation between increasing concentrations of CKB4 and decreasing amounts of CCA1 (Figure 3D). These results strongly suggest that over-expression of CKB4 does not modulate CCA1 protein accumulation. Our previous studies have shown that CKB4-MYC-ox plants exhibit increased CK2 activity that correlates with the circadian phenotypes observed in CKB4-MYC-ox plants [28]. Next we explored whether decreasing CK2 activity could alter the CCA1 protein accumulation or CCA1 repressive function. As a long-term and constant depletion of CK2 activity is lethal for plants [56], the CCA1 protein abundance was analyzed in the presence or in the absence of specific CK2 inhibitors such as DRB (5,6-dichloro-1-beta-D-ribofuranosylbenzimidazole) or DMAT (2-dimethylamino-4,5,6,7-tetrabromo-1H-benzimidazole) [57]. In a complementary approach, CCA1 protein abundance was examined in plants expressing a dominant negative CK2 alpha subunit (CKA3-) under the control of the Dexamethasone (Dex) inducible promoter [56]. Our studies showed that treatment with the inhibitors (Figure 3E) or the induction of CKA3- by Dex (Figure 3F) did not significantly alter CCA1 protein accumulation as compared with non-treated plants. In clear contrast, *TOC1:LUC* expression was markedly affected by the decreased pattern of CK2 activity. Indeed, treatment with DMAT or with DRB progressively reduced the amplitude and lengthened the period of *TOC1:LUC* expression in a dose-dependent manner (Figure 3G and Figure S5). Similar effects were observed after treatment of CKA3- plants with Dex (Figure 3H) while *TOC1:LUC* expression was not affected when WT plants were treated with Dex (not shown). This is noteworthy, as decreasing CK2 activity leads to the opposite phenotypes of those observed in CKB4-MYC-ox plants. Therefore, CK2 activity is important in controlling the circadian waveform of *TOC1:LUC* expression. As CKB4-MYC-ox reduces the severity of CCA1 repressive function on *TOC1* expression, we reasoned that decreasing CK2 activity should have the opposite effects. To explore this hypothesis, we examined *TOC1:LUC* expression in CCA1-YFP-ox/CKA3- plants before and after treatment with Dex. Our studies showed a very reduced amplitude of *TOC1:LUC* expression in CCA1-YFP-ox/CKA3- plants treated with Dex (Figure 3H). Therefore, and consistently with our hypothesis, the repressive function of CCA1 appears to be enhanced by decreasing CK2 activity. Together, these results show that CK2 and CCA1 have opposing functions in the regulation of *TOC1* expression and suggest a possible role for CK2 antagonizing CCA1 regulatory activity.

### CK2 antagonizes CCA1 binding to the promoters of its target genes

**Figure 4:**
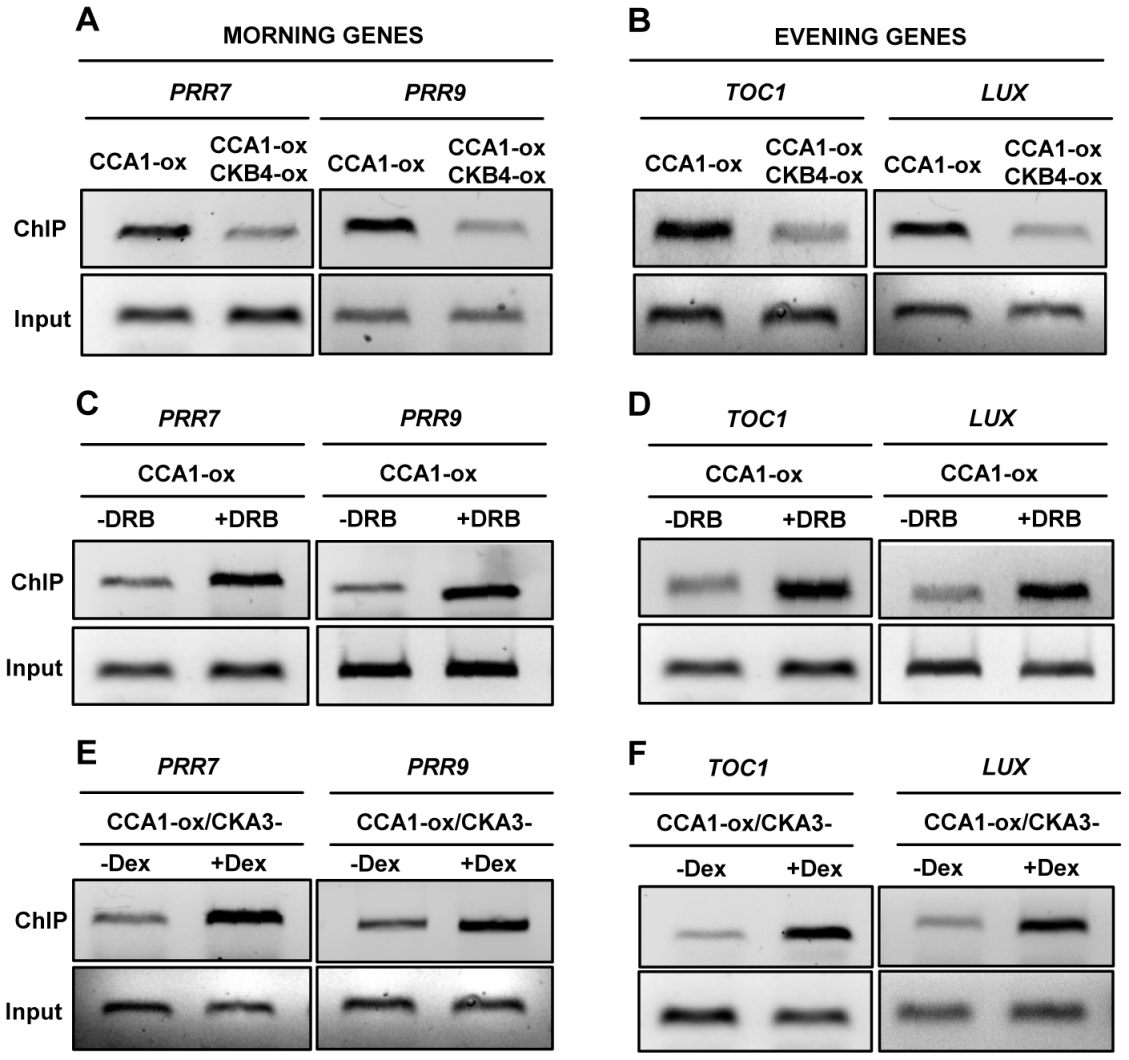
CK2 antagonizes CCA1 binding to the promoters of its target genes. ChIP analysis of CCA1 binding to the promoters of the morning-expressed genes *PRR7* and *PRR9* (A, C and E) or the evening-expressed genes *TOC1* and *LUX* (B, D and F). Analysis was performed in CCA1-YFP-ox and CCA1-YFP-ox/CKB4-MYC-ox plants (A and B) or in CCA1-YFP-ox plants collected after treatment for 48 h with 150 µM DRB (+DRB) (C and D). As a control, samples were similarly processed in the absence of DRB (-DRB). (E and F) ChIP analysis of CCA1-YFP-ox/CKA3- plants collected after induction with 1 µM Dex for 48 h (+Dex). As a control, samples were similarly processed but in the absence of Dex (-Dex). Input DNA was used as a control. Plants were synchronized under LD cycles and samples were collected at ZT3. In all cases, the experiments were performed at least three times with similar results to those shown here.

As CK2 activity reduces the severity of CCA1-YFP-ox phenotypes but this effect is not due to increased degradation of CCA1, we reasoned that CK2 might modulate CCA1 transcriptional regulatory function. To explore this possibility, we examined by chromatin immunoprecipitation (ChIP) assays, the *in vivo* binding of CCA1 to the promoters of its target genes. CCA1 was proposed to be part of a morning loop regulating the expression of both morning- and evening-expressed genes [58]. However, direct *in vivo* binding to oscillator genes was only demonstrated for *TOC1* [52]. Our ChIP assays with CCA1-YFP-ox plants confirmed the binding of CCA1 to the *TOC1* promoter (Figure S6) and also revealed the physical association of CCA1 to the promoters of the oscillator genes *PRR7*, *PRR9* and *LUX* (*LUX ARRHYTHMO*) (Figure S6). We next examined the effects of over-expressing CKB4 by comparing the binding of CCA1 in single CCA1-YFP-ox and double CCA1-YFP and CKB4-MYC over-expressing plants. Our results showed that over-expression of CKB4 considerably decreased the binding of CCA1 to the promoters of the morning-expressed genes *PRR7* and *PRR9* (Figure 4A) as well as the evening-expressed genes *TOC1* and *LUX* (Figure 4B). Conversely, decreasing CK2 activity by treatment with DRB (Figure 4C and 4D) or by inducing the dominant negative CK2 mutant (Figure 4E and 4F) had the opposite effect, with an evident increment of CCA1 binding to these promoters. Q-PCR analysis revealed the significance of the binding changes (Figure S6) with p-values<0.001 in all cases. Furthermore, DRB treatment of CCA1-YFP-ox/CKB4-MYC-ox plants significantly decreased the effects of CKB4 over-expression as compared with non-treated plants (Figure S6), suggesting that phosphorylation is indeed important for CKB4 regulation of CCA1 activity. The link between CCA1 phosphorylation and function was also reinforced in studies in which WT and *cca1-11* mutant plants [59] were treated with DRB. Our results showed that treatment of WT plants expressing the promoter of the clock-controlled gene *CAB2* (*CHLOROPHYLL A/B BINDING PROTEIN 2*, or LIGHT HARVESTING COMPLEX B1, LHCB1*1) fused to luciferase [60] considerably lengthened circadian period (Figure S7). However, the effects of DRB were considerably reduced in *cca1-11* mutant plants as compared to *cca1-11* untreated plants (Figure S7). A similar trend was observed for *TOC1:LUC* expression in *cca1-1/lhy-11* mutant plants [61], in which the up-regulation of *TOC1* by the absence of the CCA1 and LHY repressors was not importantly affected by DRB treatment (Figure S7). The alteration of *TOC1:LUC* expression in *cca1-1/lhy-11* mutant plants was more severe than the circadian phenotypes previously described [61]. This might be due to different growth conditions, different intensities of light different regimes of entrainment and/or the different reporters. Together, our results indicate that the circadian function of CK2 is mostly mediated by CCA1/LHY, which reinforces the link between these components. Our results also indicate that CK2 activity antagonizes CCA1 regulation of circadian gene expression by interfering with the binding of CCA1 to the promoters of the oscillator genes. A phosphorylation-dependent inactivation of transcriptional activity was previously reported to be important in other circadian systems [62].

### Proper regulation of CK2 activity is important for temperature compensation in Arabidopsis

Our results show that CK2 activity regulates *TOC1* expression. As *TOC1* is also modulated by temperature [40], we next explored whether CK2 regulatory functions were affected by temperature. To that end, we compared the waveforms of *TOC1:LUC* expression in WT, CKB4-MYC-ox and CCA1-YFP-ox/CKB4-MYC-ox plants synchronized for 7 days under LD cycles at 22°C and then transferred to 12°C, 22°C or 27°C. Our results showed that over-expression of CKB4 resulted in higher amplitude and slight advanced phase of *TOC1* promoter activity at 27°C (Figure S8), a phenotype slightly more severe but following the same trend than that observed at 22°C (Figure S8). Analysis of *TOC1:LUC* expression at 12°C revealed that CKB4-MYC-ox plants exhibited a lower amplitude and a slightly advanced phase compared to WT (Figure S8). The differential changes in gene expression by temperature could be fully appreciated in Figure S9 showing point-by-point comparisons of *TOC1:LUC* expression in WT and CKB4-ox at 27°C and 12°C. These results indicate that CK2 function regulating *TOC1* expression is differentially modulated by temperature. This notion was in agreement with our pharmacological studies using the CK2 inhibitor DRB. Treatment with DRB at 27°C resulted in a delayed phase and lower amplitude of *TOC1:LUC* expression whereas at 12°C, only subtle phenotypes of *TOC1:LUC* phase or amplitude were observed (Figure S10). Thus, decreasing CK2 activity has stronger effects at high temperatures. If CK2 regulation of *TOC1* expression is differentially modulated by temperature, then the CK2 activity counteracting CCA1 repressive function should become also most apparent at the higher range of temperatures. Indeed, our analysis with the double CCA1-YFP-ox/CKB4-MYC-ox plants revealed very similar waveforms of *TOC1:LUC* expression in WT and CCA1-YFP-ox/CKB4-MYC-ox plants at both 27°C and 22°C (Figure S8) suggesting that over-expression of CKB4 efficiently reverted the repressive function of CCA1 at these temperatures. However, at 12°C, the amplitude of *TOC1:LUC* expression in CCA1-YFP-ox/CKB4-MYC-ox plants was lower than in WT plants (Figure S8) suggesting that CK2 activity interfering with CCA1 repressive function is less effective or that additional mechanisms are engaged to regulate *TOC1* expression at low temperatures. We also observed an earlier declining phase, particularly evident at 27°C. The mechanism behind this phenotype might rely on the direct/indirect regulation of *TOC1* declining phase by CK2/CCA1 and/or other clock components.

**Figure 5:**
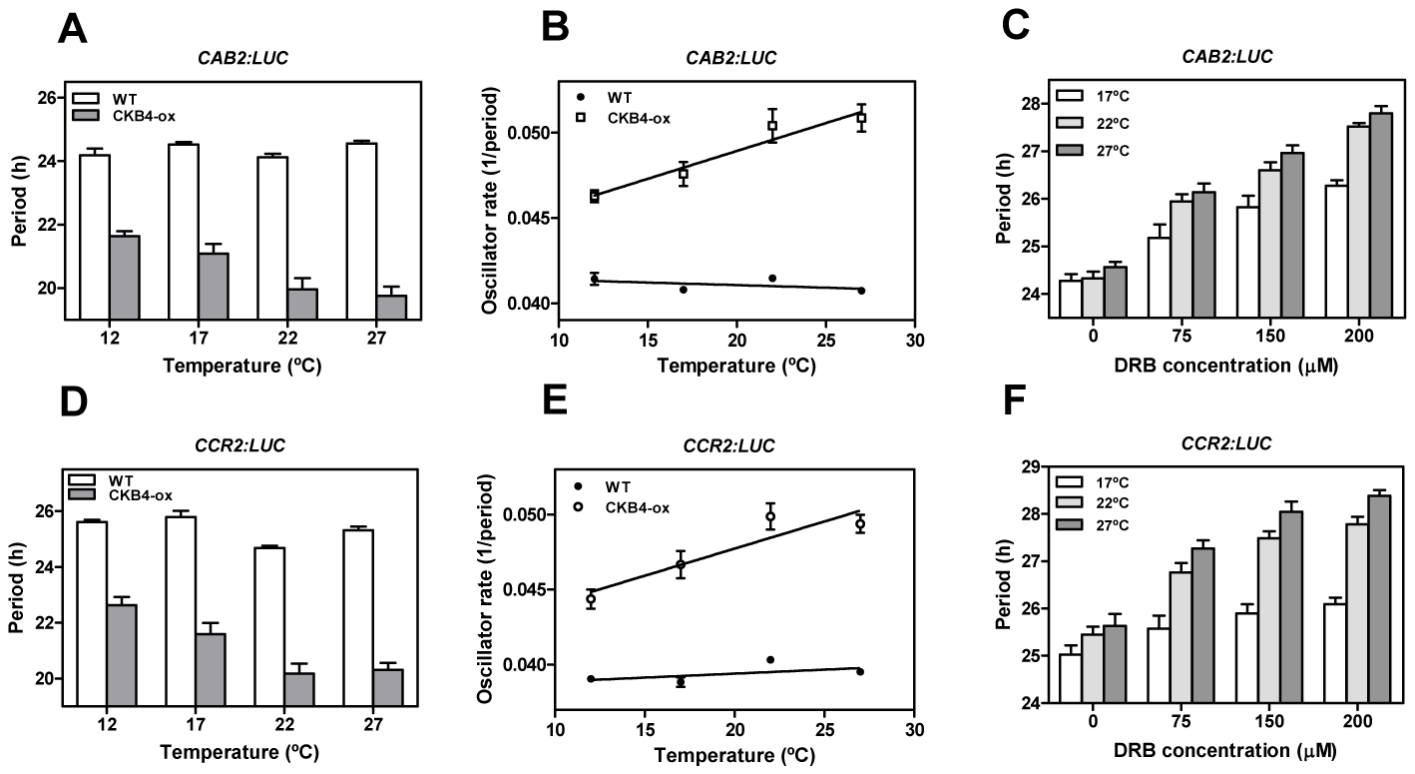
A link between CK2 and temperature compensation in Arabidopsis. Free-running periods of *CAB2:LUC* (A) and *CCR2:LUC* (D) in WT and CKB4-MYC-ox plants entrained under LD cycles at 22°C and subsequently transferred to LL conditions at 12°C, 17°C, 22°C or 27°C. Data are represented as means ± SEM of the period estimated of approximately 12 plants (from two independent experiments). The inverse of the period (oscillator rate) from *CAB2:LUC* (B) and *CCR2:LUC* (E) was used to extrapolate the linear regression of the temperature response. Free-running periods for *CAB2:LUC* (C) and *CCR2:LUC* (F) of WT plants entrained under LD cycles at 22°C and subsequently transferred to LL conditions at 17°C, 22°C and 27°C. Plants were treated with 0, 75, 150, 200 µM DRB. Data are the means ± SEM of the period estimated of approximately 12 plants.

In view of our findings and based on previous studies assigning a major role for CK2 in temperature compensation within the Neurospora circadian system [30], we next examined the possible connection between temperature compensation and CK2 activity in Arabidopsis. As the direct effects of temperature on *TOC1* expression might mask real clock period compensation, we monitored overall clock output rates by using the morning-expressed reporter *CAB2:LUC* and the evening-expressed reporter *CCR2:LUC* (*COLD-CIRCADIAN RHYTHM–RNA BINDING2*). We determined the free-running period (FRP) in plants entrained at 22°C and then transferred to constant light at 12°C, 17°C, 22°C or 27°C. As expected, our results showed that circadian clock function in WT plants was temperature compensated with similar period length at the different temperatures (Figure 5A, 5B, 5D, 5E, and Figure S11). In CKB4-MYC-ox plants, the FRP was shorter than that of WT plants at all temperatures examined. However, the period shortening for *CAB2:LUC* (Figure 5A) and *CCR2:LUC* (Figure 5D) became more severe at the higher end of the temperature range. The temperature dependency of period shortening was also evident when the inverse of the period (as an indication of the oscillator rate) was plotted against temperature (Figure 5B and 5E). The linear regression analysis showed that the slope was significantly deviated from zero (p-values<0.0001) in CKB4-MYC-ox plants but not in WT (Figure 5B and 5E). These results were in agreement with our studies using CK2 inhibitors in which the increased FRP could be correlated with increasing amounts of the inhibitor (Figure 5C and 5F). Noticeably, period lengthening was more severe at high temperatures (Figure 5C and 5F and Figure S11). Therefore, manipulation of CK2 activity by over-expression of CKB4 or by pharmacological inhibition modulates the temperature compensation profiles. Increased CK2 activity leads to under-compensated clock function (*i.e.* the clock runs faster at high temperatures) whereas decreased CK2 activity results in a slightly over-compensation (*i.e.* the clock runs slower at high temperatures). The temperature dependent function of CK2 is manifested by the different phenotypes of clock outputs at various temperatures such that period compensation is not properly achieved when CK2 activity is mis-regulated.

### Temperature modulates both CK2 and CCA1 regulatory activities

**Figure 6:**
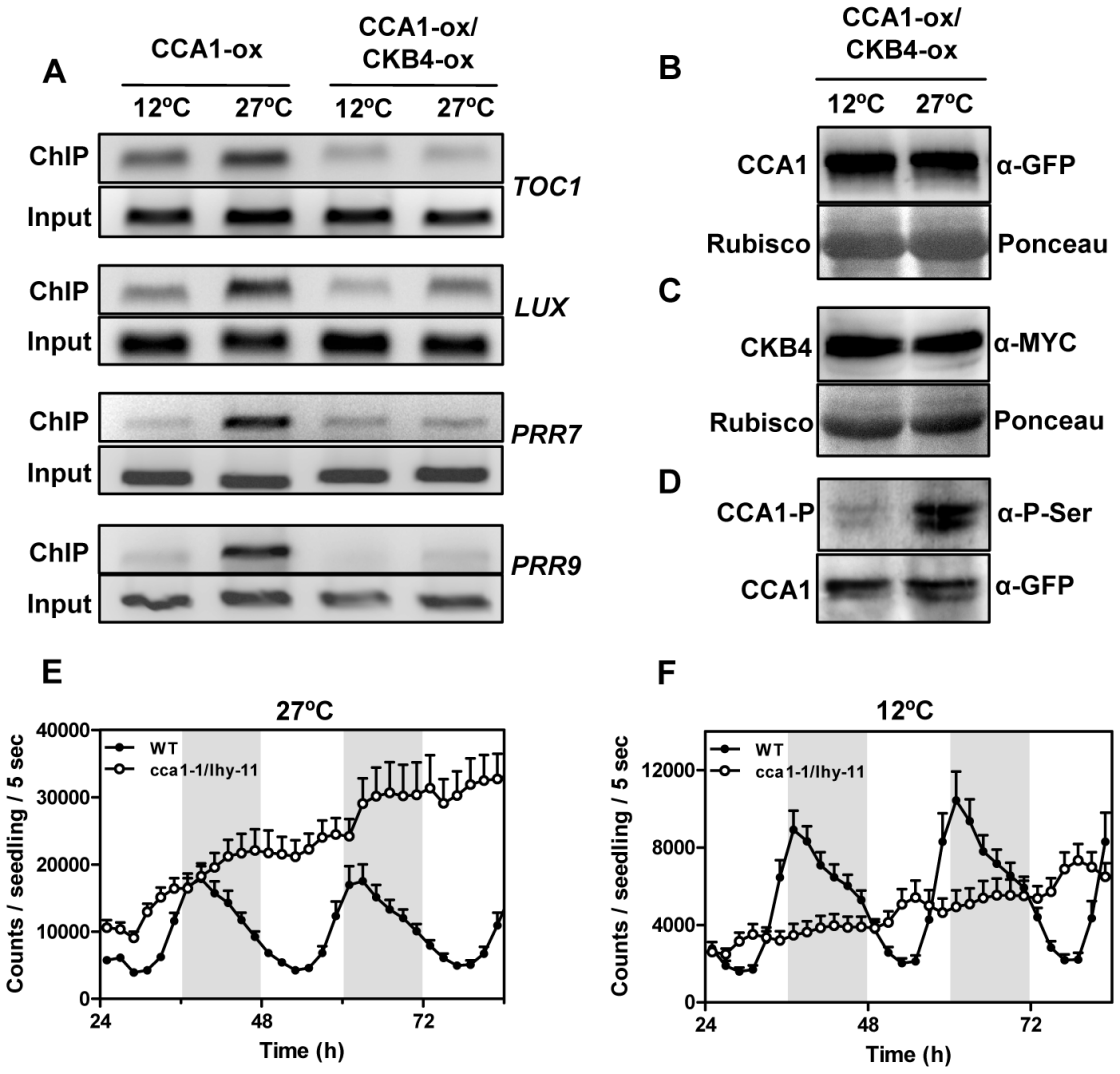
Effects of temperature and CK2 kinase function on CCA1 binding activity. (A) ChIP analysis of CCA1 binding to the promoters of *TOC1*, *LUX, PRR7* and *PRR9* in plants entrained under LD cycles at 22°C and transferred to 12°C or 27°C. Samples were collected after 35 h under LL. Input DNA was used as a control. Immunodetection of (B) CCA1-YFP and (C) CKB4-MYC protein accumulation in CCA1-YFP-ox/CKB4-MYC-ox plants grown under the same conditions described in (A). Similar protein transference in each lane was verified by staining with Red Ponceau. (D) Western-blot analysis of CCA1-YFP-ox/CKB4-MYC-ox protein extracts immunoprecipitated with anti-GFP antibody (α-GFP) with subsequent detection of phosphorylated CCA1 isoforms (CCA1-P)(α-PSer) at 12°C or 27°C. Luminescence of *TOC1:LUC* expression in WT and *cca1-1/lhy-11* plants grown under LD cycles at 22°C and subsequently transferred to 27°C (E) or 12°C (F). Plots are means ± SEM of 12 individual seedlings. The white and solid boxes correspond to the light and dark periods, respectively. The experiments were performed at least twice with similar results to those shown here.

In a last part of our study, we were interested in determining the mechanism underlying the temperature-dependent CK2 function in the Arabidopsis circadian clock. Our results indicated that CK2 interferes with the binding of CCA1 to the promoters of its target genes. Therefore, we next explored whether the temperature-dependent phenotypes of CK2 could be mechanistically linked with altered CCA1 activity. We examined by ChIP assays the CCA1 binding activity in CCA1-YFP-ox and CCA1-YFP-ox/CKB4-MYC-ox plants entrained at 22°C and then transferred to 12°C or 27°C. Q-PCR analysis of CCA1-YFP-ox plants revealed significantly increased amplification at 27°C compared to 12°C (Figure 6A and Figure S12) suggesting that CCA1 binding is regulated by temperature. Previous studies have shown that the temperature dependence of the heat capacity is a thermodynamic property of the majority of sequence-specific DNA-protein interactions [63]. Compared to CCA1-YFP-ox, a much reduced amplification was observed when CCA1-YFP-ox/CKB4-MYC-ox plants were examined (Figure 6A), with reduced differences in CCA1 binding between 12°C and 27°C (Figure S12). These results indicate a temperature-dependent regulation of both CCA1 binding activity and the CK2 antagonistic function. The differences in binding cannot be attributable to changes in CCA1 and CKB4 mRNA (not shown) or protein accumulation (Figure 6B, 6C, and Figure S12) did not vary at different temperatures. In contrast, the CCA1 phosphorylated isoforms were considerably increased at 27°C, as revealed by the immunoprecipitation assays with anti-GFP antibody followed by detection with anti-P-Ser antibody (Figure 6D). To verify that the observed effects were not due to artefactual protein over-expression, we performed ChIP assays with a *CCA1prom:CCA1-HA-YFP* line, which expresses CCA1 under its own promoter [53]. This line displays a circadian period (25.6±0.6) slightly longer than WT plants (24.3±0.1) [53]. Using this line, we confirmed the CCA1 binding to its target loci and verified a regulatory role of temperature on this binding (Figure S12). Our conclusions were also in agreement with studies of *TOC1:LUC*-expressing double mutant *cca1-1/lhy-11* plants [61]. The results showed that the luminescence signals damped high at 27°C (Figure 6E) indicating that *TOC1* repression was clearly alleviated by the absence of CCA1 and LHY. However, this function was not so evident at 12°C, with *cca1-1/lhy-11* plants displaying intermediate luminescence signals (Figure 6F). These results also suggest that at 12°C and in the absence of CCA1 and LHY, additional factors contribute to *TOC1* repression. The previously postulated major role for CCA1 at low temperatures [40] might be reflecting the decreased regulatory function of CK2 at the lower range of temperatures. In consonance with this hypothesis, our studies showed that circadian gene expression was clearly affected in CCA1-ox and in *cca1-11* mutant plants at high temperatures (Figure S13) and the DRB effects were more severe at 27°C than at 12°C (Figure S13). In agreement with these results, we observed more evident effects of DRB on CCA1 binding at high than at low temperatures (Figure S14). Altogether, our results suggest that the temperature-dependent balance between CCA1 binding activity and CK2 opposing function contributes to proper temperature compensation in Arabidopsis.

### CCA1 dephosphorylated isoforms are preferentially bound to the promoters of the oscillator genes

**Figure 7:**
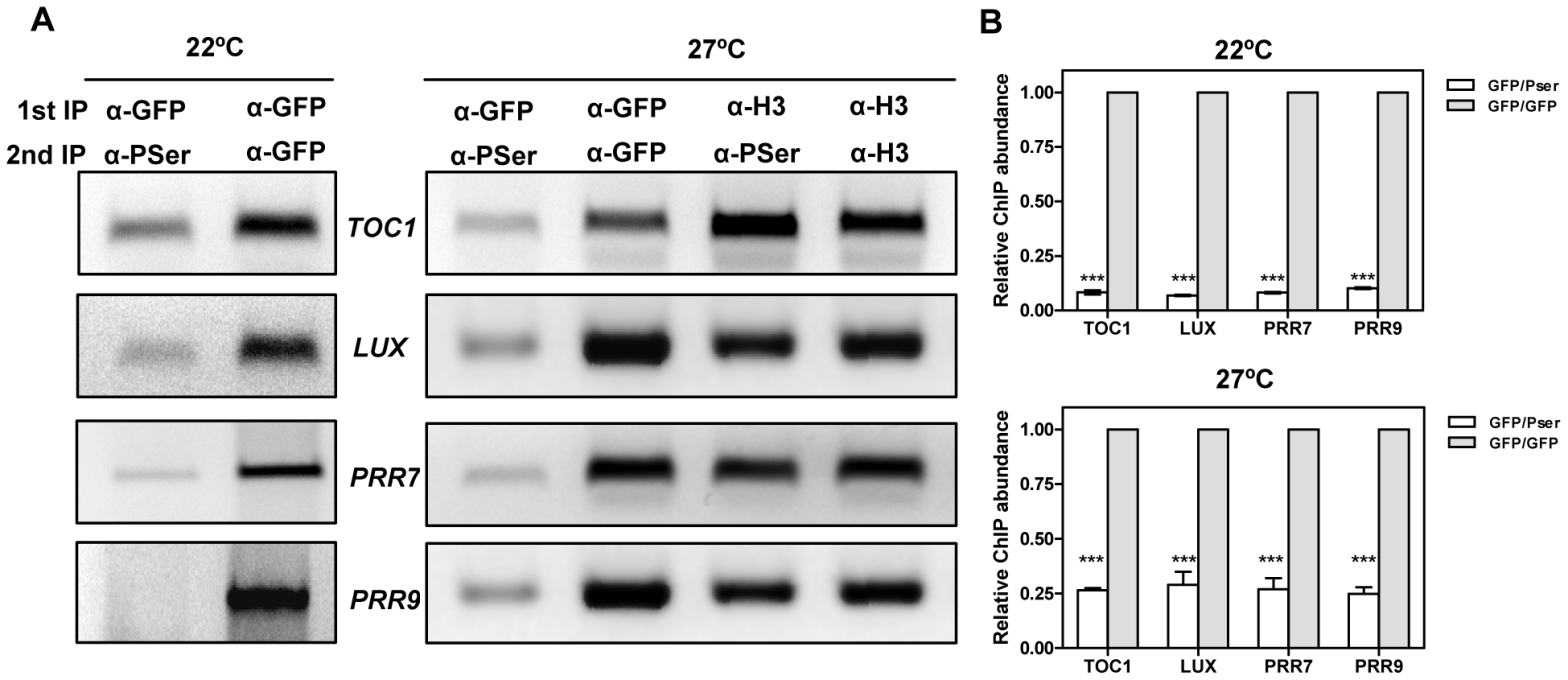
Analysis of CCA1 isoforms bound to the promoters of the oscillator genes. (A) Double ChIP assays of CCA1 isoform binding to the promoters of *TOC1*, *LUX, PRR7* and *PRR9* in CCA1-YFP-ox/CKB4-MYC-ox plants entrained under LD cycles at 22°C and transferred to LL conditions at 22°C or 27°C. Samples were taken after 35 h under LL. The first immunoprecipitation was performed with α-GFP antibody followed by a second round with α-PSer or with α-GFP. The combinations of α-H3/α-H3 antibodies or α-H3/α-Pser antibodies were used as controls. The experiments were performed at least twice with similar results to those shown here. (B) Q-PCR analysis of CCA1 isoform binding by double ChIP assays. Data are shown as means ± SD relative to the maximum value of two independent experiments (*** p-value<0.001).

Our results indicate that CK2 interferes with CCA1 function and suggest an inverse correlation between CK2 activity and CCA1 binding. We next performed Double ChIP assays at temperatures of maximal CCA1 binding (22°C and 27°C) in an attempt to estimate the phosphorylated CCA1 fraction that is associated with the promoters. We performed a double round of immunoprecipitation, firstly, with the α-GFP antibody to immunoprecipitate total CCA1 protein bound to the target promoters and secondly, with the α-PSer antibody to discriminate between phosphorylated and dephosphorylated CCA1 isoforms. By virtue of doubling-up the immunoprecipitation round, we expected to get PCR amplification only if CCA1 phosphorylated isoforms were preferentially bound to the target promoters (Figure S15). Our results showed slightly above background amplification that was observed at both 22°C or 27°C and for all the promoters examined (Figure 7A and 7B). This corresponds to only a minimal fraction of the total amount of CCA1 bound to the promoters, as assayed by double round of immunoprecipitation with the anti-GFP antibody (Figure 7A and 7B). The reliability of these results is supported by the abundant CCA1 phosphorylated isoforms observed at 27°C (Figure 6D) and the efficient detection of immunoprecipitated CCA1 by the P-Ser antibody (Figure 2A and Figure 6D). In addition, the lack of amplification in the Double ChIP assays was not likely due to reduced efficiency of the technique, as Double-ChIP experiments similarly processed with α-Histone3 (α-H3) antibody followed by α-PSer immunoprecipitation revealed clear amplified bands in all cases (Figure 7A and Figure S15). Together, the results confirmed and extend our findings suggesting that the complex regulatory interplay between CK2 and CCA1 binding activity is modulated by temperature and contributes to proper temperature compensation within the Arabidopsis circadian clock.

## Discussion

Our results show that over-expression of CKB4 reduces the severity of CCA1-YFP-ox phenotypes while the lack of additive phenotypes in CKB4-MYC-ox/*cca1-1/lhyRNAi* plants indicates that CKB4 and CCA1 function in the same signalling pathway. Repression of *TOC1* expression in CCA1-YFP-ox plants was alleviated by over-expression of CKB4, which assigns an important role for CKB4 modulating one of the main feedback loops described in Arabidopsis [58]. Pharmacological treatment with specific CK2 inhibitors or the use of plants expressing a CK2 inducible dominant mutant reflected the involvement of CK2 activity in this regulation, rather than a holoenzyme-independent function of the CK2 regulatory subunit [64] . In human cells, treatment with DRB or DMAT also results in long-period phenotypes [19]-[21]. Similarly, decreased CK2 activity causes long-period behavioral rhythms in Drosophila [22], [23]. These results highlight a remarkable conservation of CK2 circadian function among very different organisms including plants, insects and mammals.

Based on studies with other circadian systems, it was plausible to assume that phosphorylation of CCA1 by CK2 might mediate changes in CCA1 accumulation or subcellular localization [12]. However, our results assigned a role for CK2 in the control of CCA1 binding activity rather than protein accumulation or localization. Our studies also revealed that CCA1 regulates the expression of morning- and evening-expressed genes most likely by direct binding to their promoters. Therefore, by antagonizing this binding, CK2 can precisely modulate CCA1 regulation of circadian gene expression. The binding of CCA1 to the LIGHT-HARVESTING CHLOROPHYLL A/B1*3 (LHCB1*3) promoter was shown to be increased by CK2 [25], [26]. However, the authors also reported that in CKB3-ox plants, the induction of the LHCB1*1 gene was reduced compared to WT (Sugano et al., 1999). In our studies, we observed that CK2 antagonizes CCA1 binding to the promoters of the oscillator genes. This function consistently fits with the CKB and CCA1 inverse correlation between phenotypes and expression. Evidence that CK2 phosphorylation decreases protein binding to target promoters was also provided for the bZIP transcription factor HY5 [65]. Our binding analyses were consistent with the Double-ChIP results showing that CCA1 dephosphorylated isoforms were preferentially bound to the promoters of CCA1 target genes. An altered ratio of phosphorylated/dephosphorylated isoforms by increased or decreased CK2 activity is fully consistent with the circadian phenotypes observed in CKB4-MYC-ox and CCA1-YFP-ox/CKB4-MYC-ox plants. This inhibitory function is not exclusive of the plant circadian system as a phosphorylation-dependent inactivation of transcriptional function was previously reported in other circadian systems [62].

To be effective as a key mechanism of clock progression, the interplay between CK2 and CCA1 should be in turn precisely regulated. Indeed, we found that different temperatures affect CK2 regulatory function. The higher amplitude of *TOC1:LUC* expression in CKB4-MYC-ox plants was mostly observed at 22°C and 27°C while the activating function of CK2 on *TOC1* expression was not so clearly observed at 12°C. The lower amplitude of *TOC1:LUC* expression in CKB4-MYC-ox plants at 12°C is difficult to explain, but denotes that a different mechanism is engaged at low temperatures and emphasizes the complexity of the temperature response within the Arabidopsis circadian network. This notion is reinforced by the results showing that CK2 also phosphorylates LHY [26]. Thus, it is possible that the previously described role of LHY-GI in clock temperature compensation [40] might be also regulated by CK2. Notably, CCA1 binding activity appeared to be also modulated by temperature in CCA1-ox plants. Despite the lower binding at 12°C, CCA1 is still able to repress *TOC1* expression at 12°C as evidenced by our luminescence assays. Furthermore, CK2 activity might also have a residual function as it was able to interfere with CCA1 binding. Therefore, there is a parallelism between the temperature regulation of CCA1 binding and CK2 activity. We proposed that the dynamic regulation of these activities by temperature is important for clock function, as altering the functional expression by over-expression, mutation or pharmacological inhibition deregulates the temperature-dependent modulation of gene expression.

Unlike the temperature dependency of most biological and biochemical activities, the circadian clock sustains period length over a range of constantly maintained temperatures [66]. When we examined clock outputs, we found that the temperature compensation profiles were altered in CKB4-MYC-ox plants compared to those observed in WT. A simple explanation would be that CK2 activity might be compromised only when CKB4 abundance is increased above physiological limits. However, decreased CK2 activity by pharmacological inhibitors also led to altered temperature compensation. Additionally, CKB4 protein abundance did not vary between 12°C and 27°C. Together, these findings indicate that CK2 contributes to proper temperature responses in Arabidopsis: increased CK2 activity led to under-compensation whereas decreased activity resulted in a slightly over-compensated clock. This is noteworthy because previous studies in Neurospora have also implicated CK2 in temperature compensation [30]. Therefore, CK2 integrates a molecular clock component with a regulatory function that is conserved among different circadian systems. However, the specific mechanisms of CK2 function differ among organisms. In Neurospora, the stability of FRQ is controlled, at least in part, by a CK2-dependent phosphorylating mechanism, which facilitates the FRQ protein degradation preferentially at high temperatures [30]. In Arabidopsis, CK2 does not affect CCA1 protein accumulation but rather its transcriptional activity. Our results suggest that a precise temperature regulation of CK2 and CCA1 activity shapes the temperature compensation profile. Altering this delicate balance by over-expression, mutation or pharmacological inhibition affects the period compensation. In addition to the CK2-CCA1 counterbalancing loop, other loops and/or different mechanism should also account for precise regulation of temperature compensation. In fact, prior studies have proposed that LHY and GI contribute to temperature compensation in Arabidopsis [40]. Our findings focusing on CCA1 binding and CK2 activities add exciting insights into the mechanisms of temperature compensation in Arabidopsis. It would be also interesting to perform similar studies with the other three CK2 regulatory subunits in order to highlight functional similarities or divergences in the control of temperature compensation. Our findings are also in line with the notion that evolution might enhance fitness under different climate conditions without directly affecting the expression of central oscillator components but rather modulating their activity. Circadian clock adaptation to different environments might thus rely on key regulatory factors that modulate the oscillator activity. Further studies using different *Arabidopsis* ecotypes can provide new insights into how this regulation might have shaped plant adaptation to different climate areas around the globe. The parallelism in temperature dependency of CCA1 and CK2 activities and the inhibitory effect of CK2 on CCA1 function is in agreement with a previously described model for temperature compensation [33]. The model proposes that the temperature independence of circadian period by the clock occurs through the balance between two biochemical activities, each of which has a similar temperature dependency [33]. Our studies would be also in consonance with the notion that temperature compensation is not only determined by central clock components but also by other elements that function in *trans* to regulate the core proteins [40]. A recent computer modeling study has proposed that a switch-like mechanism might regulate period sensitivity through the control of two parameters that are a function of processes such as phosphorylation, ubiquitination or complex formation [67]. It would be interesting to identify a role of these regulatory processes as key modules shaping the temperature compensation profiles. Specifically for phosphorylation, studies in Neurospora [30] and mammals [68] indicate that in contrast to CK2, the activities of other kinases and clock-related phosphorylation events are temperature-insensitive. Our study showing that the CK2 activity antagonizes CCA1 function in a temperature-dependent manner highlights only one of the many aspects contributing to temperature compensation. Further analysis focusing on additional components and mechanisms would aid in our understanding of the intricate interacting networks responsible for temperature compensation in Arabidopsis and in other organisms.

## Materials and Methods

### Plant material, luminescence assays, flowering time, and hypocotyl length analysis

*Arabidopsis thaliana* seedlings were stratified at 4°C in the dark for 3 days on Murashige and Skoog agar medium supplemented with 3% sucrose and then transferred to light:dark conditions (LD, 12 h light:12 h hours dark) with 60 µmol m^−2^s^−1^ of cool white fluorescent light at 22°C. The list with the different plants and constructs used in this study is shown in Table S1 and S2. For CCA1-ox/CKB4-ox studies, lines 9 and 14 were used (see Figure S1 and Figure S2). For *cca1/lhy*/CKB4-ox studies, lines 4 and 5 were used (see Figure 3D and Figure S1). For Luminescence analyses were performed as previously described [28]. In experiments with CK2 inhibitors, one-week old plants were transferred to 96-well plates containing MS medium supplemented with the specified concentration of DRB or DMAT. Luminescence was recorded 24 hours after the seedlings were transferred to the plates. For Dexamethasone induction, 1 µM was added to each well at Zeitgeber Time 2 (ZT 2). For flowering time analysis, seeds were stratified in the dark at 4°C for 3 days on soil. Seedlings were grown under Short-Day (ShD, 8 h light:16 h dark) or Long-Day (16 h light:8 h dark) conditions with 60 µmol m^−2^s^−1^ of cool white fluorescent light at 22°C. Flowering time was scored by counting the number of days and number of leaves at the time of a 1 cm-high flower bolt. For hypocotyl length assays, seeds were stratified in the dark at 4°C for 4 days on MS medium supplemented with 3% Sucrose. Germination was induced by exposing the seeds to white light (60 µmol m^−2^ s^−1^) for 6 h followed by 18 h under darkness. Seeds were placed under ShD or LgD conditions (60 µmol m^−2^ s^−1^) or under continuous white light (LL) with the specified light intensities. Hypocotyl length was measured after seven days by using the Image J software (http://rsb.info.nih.gov/ij/).

### Western-blot and co-immunoprecipitation analysis

Western-blot assays were essentially performed as previously described [69]. Briefly, nine day-old seedlings were ground in liquid nitrogen and proteins were extracted in RIPA buffer (50 mM Tris-HCl pH 8.0, 150 mM NaCl, 1% NP-40 0.1% SDS, 0.5% Sodium Deoxycholate, 0.5% Polyvinylpolypyrrolidone (PVPP), 50 µm MG132, 10 mM NaF, 1 mM PMSF, 5 µg/ml Leupeptin, 1 µg/ml Aprotinin, 5 µg/ml Antipain, 1 µg/ml Pepstatin, 5 µg/ml Chymostatin). Protein concentration was calculated using the Bradford method (Bio-Rad) and 20-60 µg of total protein was loaded per lane. Proteins were transferred to nitrocellulose membranes and stained with Red Ponceau following standard protocols. Anti-MYC (clone 9E10; Sigma) and Anti-GFP (A11122; Invitrogen) antibodies were used to detect CKB4-MYC and CCA1-YFP proteins, respectively. Protein accumulation was quantified using the LAS-4000 imaging system (Fujifilm-GE Healthcare). In experiments with CK2 inhibitors, 48 hours before sampling, plants were transferred to medium supplemented with 150 µM of DRB, 150 µM DMAT or 1 µM Dex. For co-immunoprecipitation assays, nine day-old seedlings were ground in liquid nitrogen and proteins extracted in RIPA buffer. Extracts were incubated for 4 h at 4°C with Protein G–Sepharose beads (Amersham Biosciences) conjugated with Anti-GFP antibody. Immunocomplexes were washed 5 times with RIPA buffer followed by additional washing with PBS (Phosphate buffer saline). Immunoprecipitated proteins were eluted by adding Laemmli buffer followed by 4 min incubation at 95°C. Anti-MYC and Anti-GFP antibodies were used to detect CKB4-MYC and CCA1-YFP respectively. The anti-phosphoserine antibody (Anti-PSer, 4A3, Calbiochem) was used to detect phosphorylated isoforms of CCA1. The antibody recognizes serine-phosphorylated residues (in a positively charged amino acid context directly neighbouring the phosphoserine). For interaction of proteins expressed at endogenous levels, we used a polyclonal anti-CK2B antibody to the human CK2B regulatory subunit CK2B (CSNK2B; Abnova). Detection of CCA1 was performed with the Anti-MYC antibody.

### Two-dimensional gels and immunoblotting

For two-dimensional gels experiments, nine day-old seedlings were ground in liquid nitrogen and proteins extracted in lysis buffer (7 M Urea, 2 M Thiourea, 4% CHAPS, 18 mM Tris-HCl pH 8.0, 50 µm MG132, 10 mM NaF, 1 mM PMSF, 5 µg/ml Leupeptin, 1 µg/ml Aprotinin, 5 µg/ml Antipain, 1 µg/ml Pepstatin, 5 µg/ml Chymostatin). Protein concentration was determined using the Bradford method (Bio-Rad) and 40 µg of total protein was loaded onto immobilized pH gradient (IPG) strips (7 cm, pH 3–10, Amersham Biosciences) for the first dimension separation. Strips were rehydrated for 6 h at room temperature and the isoelectric point focusing was performed at 30 V for 6.5 h, 500 V for 1 h, 1000 V for 1 h and 5000 V for 7 h. Strips were subsequently equilibrated for 15 min with equilibration buffer I (50 mM Tris-HCl pH 8.8, 6 M Urea, 30% Glycerol, 2% SDS, 10 mg/ml DTT) followed by a 15 min wash with equilibration buffer II (50 mM Tris-HCl pH 8.8, 6 M Urea, 30% Glycerol, 2% SDS, 25 mg/ml Iodoacetamide). For the second dimension, strips were loaded onto SDS-PAGE 8% polyacrylamide gels followed by blotting to nitrocellulose membranes. Anti-GFP antibody was used to detect CCA1-YFP protein.

### Bimolecular Fluorescence Complementation (BiFC) and confocal microscopy

Plants over-expressing both the CCA1 protein fused to the N-terminal fragment of the Yellow Fluorescent Protein (YFP) (nucleotides: 1–462) and the CKB4 protein fused to the C-terminal part of the YFP protein (nucleotides: 463–741) were grown on MS-agar medium supplemented with 3% Sucrose under LD cycles. Fluorescence signals of hypocotyl cells were imaged using an Olympus Fluoview FV1000 confocal microscope using a 515 nm argon excitation laser. CKB4-YFP-ox, CCA1-YFP-ox, TOC1-YFP-ox, CCA1-ox/CKB4-YFP-ox, CCA1-YFP-ox/CKB4-MYC-ox, TOC1-nYFP-ox/CKB4-cYFP-ox were similarly imaged.

### ChIP and double-ChIP assays

ChIP assays were performed essentially as previously described [52]. Briefly, fourteen day-old seedlings were fixed in fixation buffer (0.4 M Sucrose, 10 mM Tris-HCl pH 8.0, 1 mM EDTA,1 mM PMSF, 1% Formaldehyde, 0.05% Triton X-100) for 10 min, followed by addition of Glycine 0.125 M and vacuum incubation during 10 min. Seedlings were subsequently ground in liquid nitrogen and extracted in extraction buffer I (0.4 M Sucrose, 10 mM Tris-HCl pH 8.0, 5 mM β-mercaptoethanol, 1 mM PMSF, 5 µg/ml Leupeptin, 1 µg/ml Aprotinin, 5 µg/ml Antipain, 1 µg/ml Pepstatin, 5 µg/ml Chymostatin and 50 µm MG132). Nuclei were then purified by centrifugation and washed with extraction buffer II (0.25 M Sucrose, 10 mM Tris-HCl pH 8.0, 10 mM MgCl_2_, 1% Triton X-100, 5 mM β-mercaptoethanol, 1 mM PMSF, 5 µg/ml Leupeptin, 1 µg/ml Aprotinin, 5 µg/ml Antipain, 1 µg/ml Pepstatin, 5 µg/ml Chymostatin and 50 µm MG132). Nuclei were resuspended in nuclei lysis buffer (50 mM Tris-HCl pH 8.0, 10 mM EDTA, 1% SDS, 5 µg/ml Leupeptin, 1 µg/ml Aprotinin, 5 µg/ml Antipain, 1 µg/ml Pepstatin, 5 µg/ml Chymostatin and 50 µm MG132). Chromatin was sonicated to approximately 500–1000 bp fragments with a sonicator (Branch). After centrifugation, soluble chromatin was diluted in ChIP dilution buffer (15 mM Tris-HCl pH 8.0, 150 mM NaCl, 1% Triton-X-100, 1 mM EDTA, 1 mM PMSF, 5 µg/ml Leupeptin, 1 µg/ml Aprotinin, 5 µg/ml Antipain, 1 µg/ml Pepstatin, 5 µg/ml Chymostatin and 50 µm MG132) and incubated overnight at 4°C with Protein G–Sepharose beads (Amersham Biosciences) conjugated with Anti-GFP antibody. Immunocomplexes were washed with low salt buffer (20 mM Tris-HCl pH 8.0, 150 mM NaCl, 1% Triton X-100, 0.1% SDS, 2 mM EDTA), high salt buffer (20 mM Tris-HCl pH 8.0, 500 mM NaCl, 1% Triton X-100, 0.1% SDS, 2 mM EDTA), LiCl wash buffer (10 mM Tris-HCl pH 8.0, 0.25 M LiCl, 1% NP-40, 1% Sodium Deoxycholate, 1 mM EDTA) and 2x TE buffer (10 mM Tris-HCl pH 8.0, 1 mM EDTA). Immunocomplexes were eluted with 1% SDS and 0.1 M NaHCO_3_ followed by overnight reverse cross-link at 65°C and proteinase K treatment for 1 h at 45°C. Immunoprecipitated DNA was isolated using the QIAquick kit (Qiagen) following the manufacturer instructions. ChIP samples were amplified by PCR, stained with SYBR Green (Molecular Probes) and resolved by electrophoresis on 2% agarose gel. Images were captured with the LAS-4000 imaging system (Fujifilm-GE Healthcare). ChIPs were quantified by Q-PCR analysis using a 96-well Lightcycler 480 system (Roche) with the Lightcycler 480 software (Version 1.5.0.39, Roche). Melting peak analysis using the LightCycler 480 Basic software module (Roche) and gel electrophoresis confirmed that primer-dimers or other non-specific products were not present. Crossing point (Cp) calculation was used for quantification using the Absolute Quantification analysis by the 2^nd^ Derivative Maximun method (LightCycler 480 Basic software module, Roche). ChIP values for each set of primers were normalized to Input values. Primers were designed using the PrimerExpress 2.0 software (Applied Biosystems) with lengths of 18-25 nucleotides, PCR amplicon lengths of 80 to 180 bp, 40-60% G:C content and melting point of 58-62°C. The list of primers used for promoter (prom) amplification and for Q-PCR analysis is shown in Table S3. In experiments with CK2 inhibitors or with plants expressing the CK2 inducible mutant, 48 hours before sampling, plants were transferred to medium supplemented with 150 µM of DRB, 150 µM DMAT or 1 µM Dex. Double-ChIP assays [70] were performed following the ChIP procedure for chromatin extraction and immunoprecipitation followed by one wash with low salt buffer (20 mM Tris-HCl pH 8.0, 150 mM NaCl, 1% Triton X-100, 0.1% SDS and 2 mM EDTA) and two washes with TE buffer (10 mM Tris-HCl pH 8.0, 1 mM EDTA). Immunocomplexes were eluted using 10 mM DTT and incubating at 37°C for 30 min. Chromatin was diluted in 40 volumes of Double-ChIP buffer (15 mM Tris-HCl pH 8.0, 150 mM NaCl, 1% Triton-X-100, 1 mM EDTA, 1 mM PMSF, 5 µg/ml Leupeptin, 1 µg/ml Aprotinin, 5 µg/ml Antipain, 1 µg/ml Pepstatin, 5 µg/ml Chymostatin and 50 µm MG132) and incubated overnight at 4°C with Protein G–Sepharose beads (Amersham Biosciences) conjugated with Anti-PSer or Anti-GFP antibody. Immunocomplexes were washed, eluted, purified and amplified as described for the ChIP protocol.

### Statistical analysis

Statistical analyses were performed using the GraphPad Prism software (GraphPad Software, Inc). For hypocotyl length and flowering time experiments, two-tailed t-tests with 99% of confidence were performed. For multiple comparisons, two-way ANOVA followed by Bonferroni post-tests were performed; photoperiod and genotype were considered as variables. For ChIP quantifications, two-way ANOVA tests followed by Bonferroni post-tests were used. Gene and genotype or temperature and genotype were considered as variables. GraphPad Prism software was also used to extrapolate the linear regression of the temperature response. Profiles for bioluminescence experiments, the estimated period length was determined by Fast Fourier Transform/Nonlinear Least Squares method (FFT/NLLS) [71] using a window of 96 hr of data excluding the first 24 hr to avoid any transient effects after transferring to constant conditions.

## Supporting Information

Figure S1 CKB4 and CCA1 expression in different CCA1-ox/CKB4-ox and *cca1/lhy/*CKB4-ox lines. (A) RT-PCR analysis of *CKB4*, *CCA1* and *ACTIN2* (*ACT2*) expression in WT and double CCA1 and CKB4 over-expressing plants. Lines 14 and 9 were used for subsequent studies. (B) RT-PCR analysis of *CKB4* and *ACTIN2* (*ACT2*) expression in WT and in *cca1-1/lhyRNAi*/CKB4-ox plants. Lines 5 and 4 were used for subsequent studies. Seedlings were entrained under LD cycles and samples were collected at Zeitgeber Time 2 (ZT2). (C) Immunodetection of CKB4 protein accumulation in *cca1-1/lhyRNAi*/CKB4-ox plants. CKB4 protein was detected using the α-MYC antibody. Seedlings were entrained under LD cycles and samples were collected at ZT2.
(0.21 MB PDF)

Figure S2 Analysis of CCA1 and CKB4 genetic interaction. Flowering time of WT, *cca1-1/lhyRNAi*, *cca1-1/lhyRNAi*/CKB4-MYC-ox and CKB4-MYC-ox plants grown under (A, B) Long-Day (LgD, 16 h light:8 h dark) or (C, D) Short-Day (ShD, 8 h light:16 h dark) conditions. Flowering time was measured as the number of leaves at flowering or the number of days to flowering (1-cm-high bolt). Data are shown as means ± SEM of three independent experiments. Similar results were obtained when flowering time was examined in *cca1-1/lhy-11* and *cca1-1/lhy-11*/CKB4-MYC-ox plants.
(0.12 MB PDF)

Figure S3 Analysis of *TOC1:LUC* diurnal expression in different genetic backgrounds. *TOC1:LUC* luminescence in seedlings maintained under LD (12 h light:12 h dark) cycles. Plots represent means ± SEM of at least 12 individual seedlings. The white and solid boxes correspond to the light and dark periods, respectively. The experiment was performed three times with similar results to those shown here.
(0.09 MB PDF)

Figure S4 Analysis of CCA1 and CKB4 molecular interaction. (A) Western-blot analysis of WT, CCA1-ox and CCA1prom:CCA1-MYC/*cca1-1* plants using an antibody to the human CK2B subunit (α-CKB). The antibody efficiently recognizes the CK2 regulatory subunits of Arabidopsis. (B) Western-blot analysis of Co-IP experiments with plants expressing CCA1 under its own promoter (CCA1prom:CCA1-MYC/*cca1-1*). Protein extracts were immunoprecipitated with the α-CKB antibody followed by detection with the α-MYC antibody. Plants were grown under LD conditions and samples were collected at ZT1.5. Nuclear localization analysis by confocal microscopy at ZT11 of plants expressing (C) CCA1-ox/CKB4-YFP-ox, (D) CCA1-YFP-ox/CKB4-MYC-ox, (E) TOC1 fused to the N-terminal fragment of YFP (TOC1-nYFP-ox) and CKB4 fused to the C-terminal fragment of YFP (CKB4-cYFP-ox) or (F) TOC1 fused to full-length YFP (TOC1-YFP-ox).
(0.40 MB PDF)

Figure S5 Effects of the CK2 inhibitors DRB and DMAT on *TOC1:LUC* expression. (A) Free-running periods estimated from *TOC1:LUC* luminescence signals in WT plants under LL conditions treated with increasing concentrations of DRB. Period was estimated from individual seedlings plotted against their relative amplitude errors. (B) *TOC1:LUC* luminescence in WT plants treated with increasing concentrations of DMAT. Luminescence was recorded under LD (12 h light:12 h dark) cycles. Data are represented as means ± SEM of luminescence signals from at least 12 independent plants.
(0.11 MB PDF)

Figure S6 Effects of CK2 activity on the *in vivo* CCA1 binding to the promoters of the oscillator genes. (A) ChIP analysis of CCA1 binding to the promoters of the morning-expressed genes, *PRR7* and *PRR9* and the evening-expressed genes *TOC1* and *LUX*. Analysis was performed with CCA1-YFP-ox plants entrained under LD cycles and samples were collected at ZT3 and ZT15. The binding regions of *TOC1, LUX* and *PRR9* promoters contain the Evening Element (EE) motif. No conserved motifs were identified in the CCA1 binding region of *PRR7* promoters. No amplification was obtained with the promoter of a clock-unrelated gene (At5g55840) or when samples were similarly processed in the absence of antibody. The experiments were performed three times with similar results to those shown here. Q-PCR analysis of CCA1 binding in (B) CCA1-YFP-ox and CCA1-YFP-ox/CKB4-MYC-ox plants; in (C) CCA1-YFP-ox with 150 µM of the CK2 inhibitor DRB; in (D) CKA3-/CCA1-YFP-ox plants induced with 1 µM Dexamethasone (Dex) or in (E) CCA1-YFP-ox/CKB4-MYC-ox plants treated with 150 µM of the CK2 inhibitor DRB. Seedlings were grown under LD cycles and collected at ZT3. Data are presented as means ± SEM relative to the input and to the maximum value of at least three independent experiments (*** p-value< 0.001).
(0.20 MB PDF)

Figure S7 Effects of temperature and CK2 kinase function on circadian gene expression. Bioluminescence of *CAB2:LUC* expression in WT (A) and *cca1-11* mutant (B) plants in the presence or in the absence of 100 µM DRB. Luminescence was measured in plants entrained under LD conditions at 22°C and transferred to LL conditions. Plots are means ± SEM of 8-12 individual seedlings. (C) Analysis of circadian period length of *CAB2::LUC* expression in WT and *cca1-11* mutant plants in the presence or in the absence of DRB. Estimated period length was determined as described in Supplemental experimental procedures. (D) *TOC1:LUC* luminescence in WT and *cca1-1/lhy-11* double mutant plants in the presence or in the absence of 100 µM DRB. Luminescence was measured in plants entrained under LD conditions at 22°C and transferred to LL conditions. Plots are means ± SEM of 8-12 individual seedlings. The experiments were repeated at least twice with similar results to those shown here.
(0.14 MB PDF)

Figure S8 Analysis of *TOC1:LUC* diurnal expression under different temperatures. Luminescence analysis of *TOC1:LUC* expression in plants entrained under LD cycles at 22°C and subsequently transferred to LD cycles at 27°C (A, D), 22°C (B, E) or 12°C (C, F). Data is shown as means ± SEM of at least 12 individual seedlings. The experiments were performed at least twice with similar results to those shown here.
(0.13 MB PDF)

Figure S9 Analysis of *TOC1:LUC* diurnal expression in CKB4-ox plants under different temperatures. Point-by-point luminescence analysis of *TOC1:LUC* expression in plants entrained under LD cycles at 22°C and subsequently transferred to LD cycles at 27°C (A) or 12°C (B). Data is shown as means ± SEM of at least 12 individual seedlings. The experiments were performed at least twice with similar results to those shown here.
(0.13 MB PDF)

Figure S10 Effects of DRB treatment on *TOC1:LUC* diurnal expression at different temperatures. *TOC1:LUC* luminescence in WT plants in the presence or in the absence of 100 µM DRB at 27°C or 12°C. Luminescence was measured in plants entrained under LD conditions at 22°C and transferred to LD cycles at 27°C (A) or 12°C (B). Plots are means ± SEM of 12 individual seedlings. The experiments were repeated at least twice with similar results to those shown here. The white and solid boxes correspond to the light and dark periods, respectively.
(0.11 MB PDF)

Figure S11 Effects of temperature and CK2 kinase function on the free-running period of the clock-output *CAB2:LUC*. Free-running periods estimated from *CAB2:LUC* luminescence signals in WT, CKB4-MYC-ox and WT plants treated with 100 µM DRB at 12°C (A), 22°C (B) and 27°C (C). Period was estimated from individual seedlings plotted against their relative amplitude errors. Data come from two independent experiments with approximately 6-12 plants per phenotype or treatment.
(0.12 MB PDF)

Figure S12 Effects of temperature and CK2 kinase function on CCA1 binding to the promoters of the oscillator genes. Q-PCR analysis of CCA1 binding to *TOC1* (A), *LUX* (B), *PRR7* (C) or *PRR9* (D) promoters. CCA1-YFP-ox and CCA1-YFP-ox/CKB4-MYC-ox plants were grown under LD cycles at 22°C and transferred to continuous light (LL) at 12°C or 27°C. Samples were collected after 35 h under LL conditions. Data are presented as means ± SD relative to the input and to the maximum value of two independent experiments. (E) Q-PCR analysis of CCA1 binding to *TOC1, LUX, PRR7*, *PRR9* and a clock unrelated gene (At5g55840) in plants expressing CCA1 under its own promoter (CCA1pro:CCA1-HA-YFP). Plants were grown under LD cycles at 22°C and transferred to continuous light (LL) at 12°C or 27°C. Samples were collected after 49.5 h under LL conditions. (F) Western-blot analysis of CCA1 protein accumulation in CCA1-YFP-ox plants at 12°C and 27°C. Similar protein transference in each lane was verified by staining with Red Ponceau. The experiments were performed twice with similar results to those shown here (** p-value<0.01; *** p-value<0.001).
(0.17 MB PDF)

Figure S13 A role for CCA1 regulating circadian gene expression at high temperatures. *TOC1:LUC* luminescence in WT and CCA1-YFP-ox plants at 27°C (A) or 12°C (B). *TOC1:LUC* luminescence in WT and CCA1-YFP-ox plants at 27°C (C) or 12°C (D) in the presence or in the absence of 100 µM DRB. (E) *CAB2:LUC* luminescence in WT and *cca1-11* mutant plants at 27°C and in the presence or in the absence of 100 µM DRB (F). Luminescence was measured in plants entrained under LD conditions at 22°C and transferred to LL conditions at the indicated temperatures. Plots are means ± SEM of 12 individual seedlings. The experiments were repeated at least twice with similar results to those shown here.
(0.15 MB PDF)

Figure S14 Effects of temperature and CK2 kinase function on CCA1 binding to the promoters of the oscillator genes. Q-PCR analysis of CCA1 binding to *TOC1, LUX, PRR7* and *PRR9* promoters in plants over-expressing CCA1 in the absence (-) or in the presence (+) of 150 µM of the CK2 inhibitor DRB. Plants were grown under LD cycles at 22°C and transferred to continuous light (LL) at 12°C (A) or 27°C (B). Data are presented as means ± SD relative to the input and to the maximum value. Samples were collected after 50 h under LL conditions.
(0.11 MB PDF)

Figure S15 Double ChIP analysis. (A) Schematic representation depicting a summary of the Double-ChIP assay described in Figure 7. A double round of immunoprecipitation (IP) was performed. In the first round, the α-GFP antibody was used to detect total CCA1-YFP protein bound to chromatin. In a second round, the α-PSer antibody was used to specifically detect the phosphorylated isoforms of CCA1 (P). PCR amplification would be obtained only if the phosphorylated CCA1 isoforms are preferentially bound to chromatin. (B) Double-ChIP assays with the combination of α-H3/anti-PSer or α-H3/anti-H3 antibodies Data are presented as means ± SD relative to the maximum value of two independent experiments.
(0.13 MB PDF)

Table S1 Plant Material used in this study.
(0.07 MB DOC)

Table S2 Constructs used in this study.
(0.05 MB DOC)

Table S3 Primers used in this study.
(0.05 MB DOC)
