# Supplementary figures and images for: The Functional Interplay between Protein Kinase CK2 and CCA1 Transcriptional Activity Is Essential for Clock Temperature Compensation in Arabidopsis
Source: PLoS Genet. 2010 Nov 4;6(11):e1001201. doi: 10.1371/journal.pgen.1001201 (PMC2973838; doi:10.1371/journal.pgen.1001201)

Figure S1

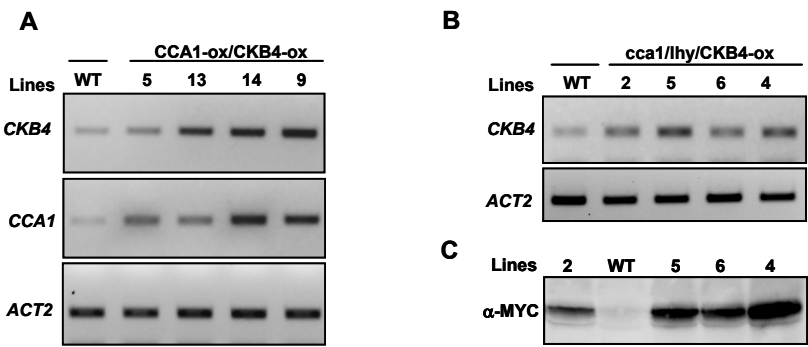

Supplement: Figure S1 — CKB4 and CCA1 expression in different CCA1-ox/CKB4-ox and cca1/lhy/CKB4-ox lines. (A) RT-PCR analysis of CKB4, CCA1 and ACTIN2 (ACT2) expression in WT and double CCA1 and CKB4 over-expressing plants. Lines 14 and 9 were used for subsequent studies. (B) RT-PCR analysis of CKB4 and ACTIN2 (ACT2) expression in WT and in cca1-1/lhyRNAi/CKB4-ox plants. Lines 5 and 4 were used for subsequent studies. Seedlings were entrained under LD cycles and samples were collected at Zeitgeber Time 2 (ZT2). (C) Immunodetection of CKB4 protein accumulation in cca1-1/lhyRNAi/CKB4-ox plants. CKB4 protein was detected using the α-MYC antibody. Seedlings were entrained under LD cycles and samples were collected at ZT2. (0.21 MB PDF) [file pgen.1001201.s001.pdf]

Figure S2

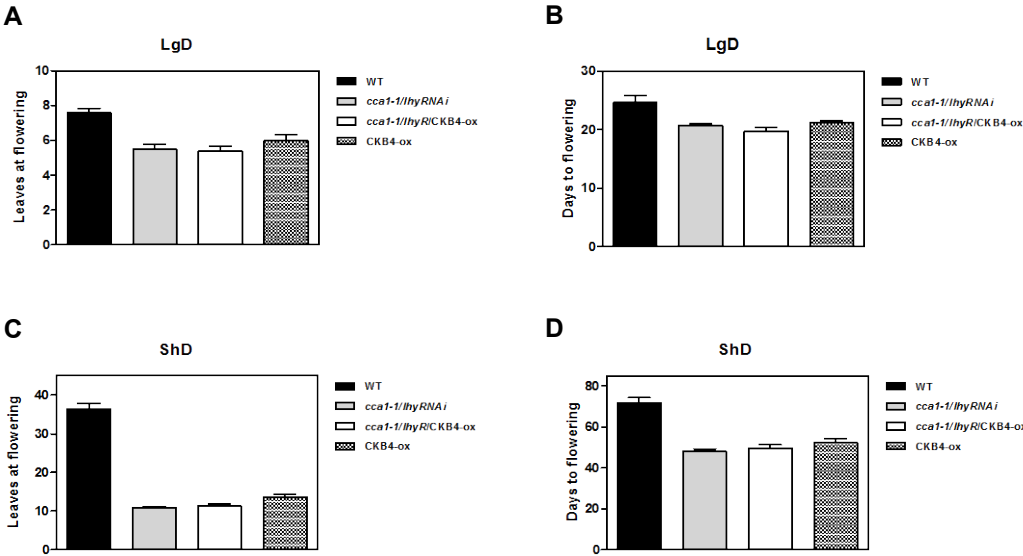

Supplement: Figure S2 — Analysis of CCA1 and CKB4 genetic interaction. Flowering time of WT, cca1-1/lhyRNAi, cca1-1/lhyRNAi/CKB4-MYC-ox and CKB4-MYC-ox plants grown under (A, B) Long-Day (LgD, 16 h light:8 h dark) or (C, D) Short-Day (ShD, 8 h light:16 h dark) conditions. Flowering time was measured as the number of leaves at flowering or the number of days to flowering (1-cm-high bolt). Data are shown as means ± SEM of three independent experiments. Similar results were obtained when flowering time was examined in cca1-1/lhy-11 and cca1-1/lhy-11/CKB4-MYC-ox plants. (0.12 MB PDF) [file pgen.1001201.s002.pdf]

**Figure S3**

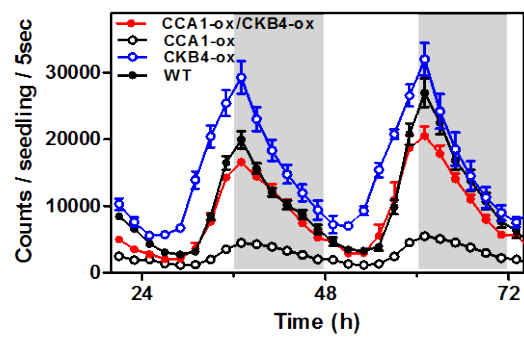

Supplement: Figure S3 — Analysis of TOC1:LUC diurnal expression in different genetic backgrounds. TOC1:LUC luminescence in seedlings maintained under LD (12 h light:12 h dark) cycles. Plots represent means ± SEM of at least 12 individual seedlings. The white and solid boxes correspond to the light and dark periods, respectively. The experiment was performed three times with similar results to those shown here. (0.09 MB PDF) [file pgen.1001201.s003.pdf]

Figure S4

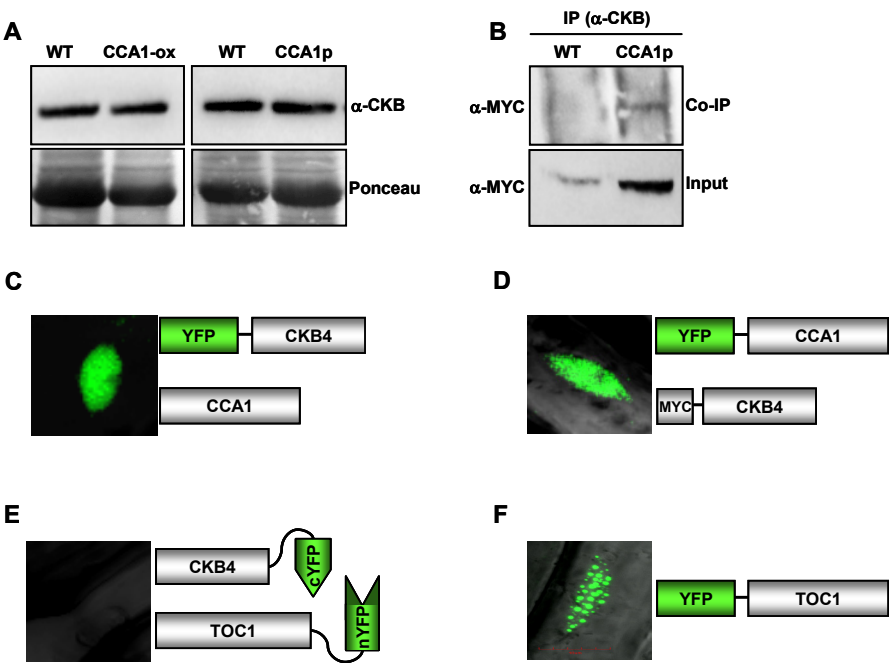

Supplement: Figure S4 — Analysis of CCA1 and CKB4 molecular interaction. (A) Western-blot analysis of WT, CCA1-ox and CCA1prom:CCA1-MYC/cca1-1 plants using an antibody to the human CK2B subunit (α-CKB). The antibody efficiently recognizes the CK2 regulatory subunits of Arabidopsis. (B) Western-blot analysis of Co-IP experiments with plants expressing CCA1 under its own promoter (CCA1prom:CCA1-MYC/cca1-1). Protein extracts were immunoprecipitated with the α-CKB antibody followed by detection with the α-MYC antibody. Plants were grown under LD conditions and samples were collected at ZT1.5. Nuclear localization analysis by confocal microscopy at ZT11 of plants expressing (C) CCA1-ox/CKB4-YFP-ox, (D) CCA1-YFP-ox/CKB4-MYC-ox, (E) TOC1 fused to the N-terminal fragment of YFP (TOC1-nYFP-ox) and CKB4 fused to the C-terminal fragment of YFP (CKB4-cYFP-ox) or (F) TOC1 fused to full-length YFP (TOC1-YFP-ox). (0.40 MB PDF) [file pgen.1001201.s004.pdf]

Figure S5

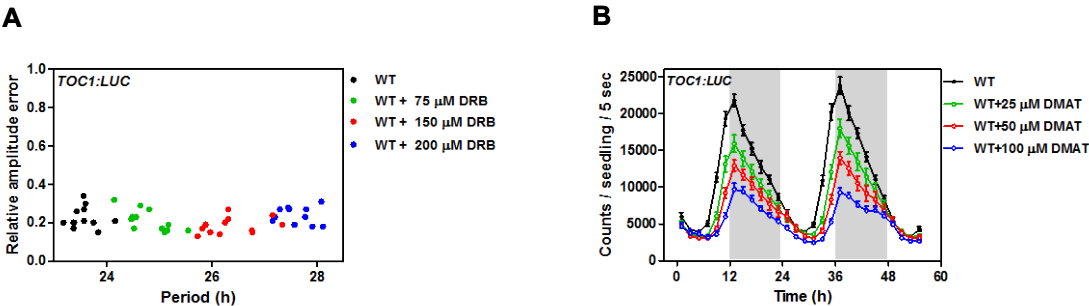

Supplement: Figure S5 — Effects of the CK2 inhibitors DRB and DMAT on TOC1:LUC expression. (A) Free-running periods estimated from TOC1:LUC luminescence signals in WT plants under LL conditions treated with increasing concentrations of DRB. Period was estimated from individual seedlings plotted against their relative amplitude errors. (B) TOC1:LUC luminescence in WT plants treated with increasing concentrations of DMAT. Luminescence was recorded under LD (12 h light:12 h dark) cycles. Data are represented as means ± SEM of luminescence signals from at least 12 independent plants. (0.11 MB PDF) [file pgen.1001201.s005.pdf]

Figure S6

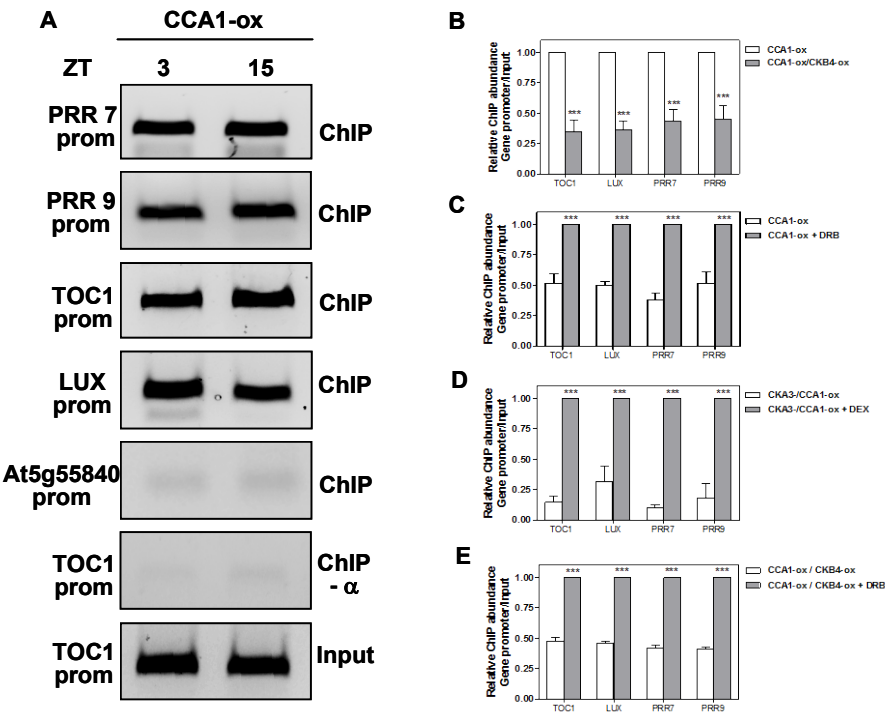

Supplement: Figure S6 — Effects of CK2 activity on the in vivo CCA1 binding to the promoters of the oscillator genes. (A) ChIP analysis of CCA1 binding to the promoters of the morning-expressed genes, PRR7 and PRR9 and the evening-expressed genes TOC1 and LUX. Analysis was performed with CCA1-YFP-ox plants entrained under LD cycles and samples were collected at ZT3 and ZT15. The binding regions of TOC1, LUX and PRR9 promoters contain the Evening Element (EE) motif. No conserved motifs were identified in the CCA1 binding region of PRR7 promoters. No amplification was obtained with the promoter of a clock-unrelated gene (At5g55840) or when samples were similarly processed in the absence of antibody. The experiments were performed three times with similar results to those shown here. Q-PCR analysis of CCA1 binding in (B) CCA1-YFP-ox and CCA1-YFP-ox/CKB4-MYC-ox plants; in (C) CCA1-YFP-ox with 150 µM of the CK2 inhibitor DRB; in (D) CKA3-/CCA1-YFP-ox plants induced with 1 µM Dexamethasone (Dex) or in (E) CCA1-YFP-ox/CKB4-MYC-ox plants treated with 150 µM of the CK2 inhibitor DRB. Seedlings were grown under LD cycles and collected at ZT3. Data are presented as means ± SEM relative to the input and to the maximum value of at least three independent experiments (*** p-value< 0.001). (0.20 MB PDF) [file pgen.1001201.s006.pdf]

Figure S7

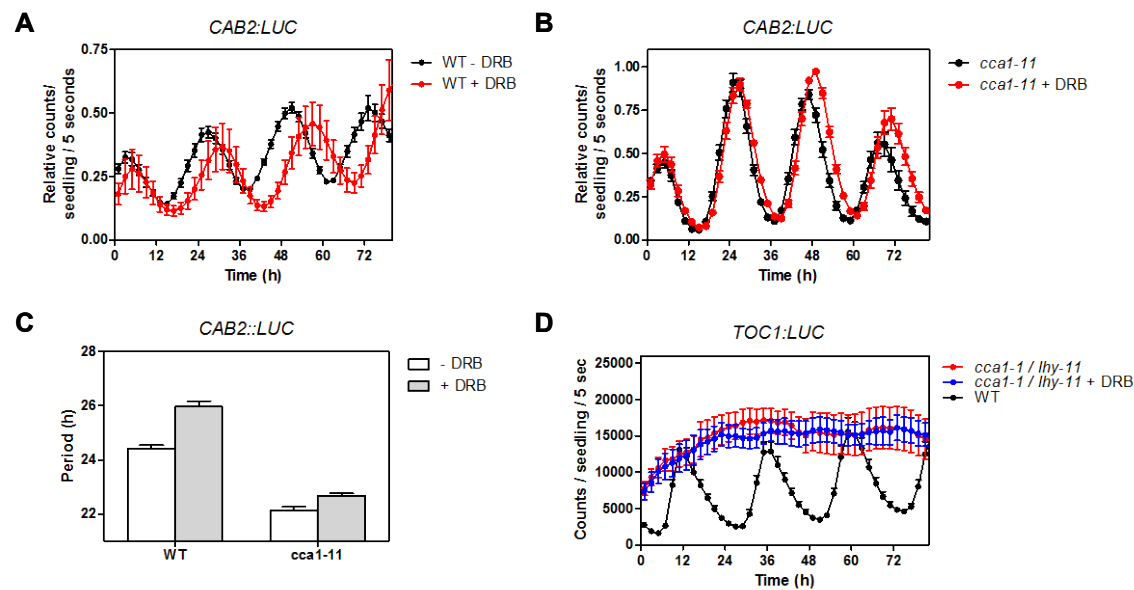

Supplement: Figure S7 — Effects of temperature and CK2 kinase function on circadian gene expression. Bioluminescence of CAB2:LUC expression in WT (A) and cca1-11 mutant (B) plants in the presence or in the absence of 100 µM DRB. Luminescence was measured in plants entrained under LD conditions at 22°C and transferred to LL conditions. Plots are means ± SEM of 8-12 individual seedlings. (C) Analysis of circadian period length of CAB2::LUC expression in WT and cca1-11 mutant plants in the presence or in the absence of DRB. Estimated period length was determined as described in Supplemental experimental procedures. (D) TOC1:LUC luminescence in WT and cca1-1/lhy-11 double mutant plants in the presence or in the absence of 100 µM DRB. Luminescence was measured in plants entrained under LD conditions at 22°C and transferred to LL conditions. Plots are means ± SEM of 8-12 individual seedlings. The experiments were repeated at least twice with similar results to those shown here. (0.14 MB PDF) [file pgen.1001201.s007.pdf]

Figure S8

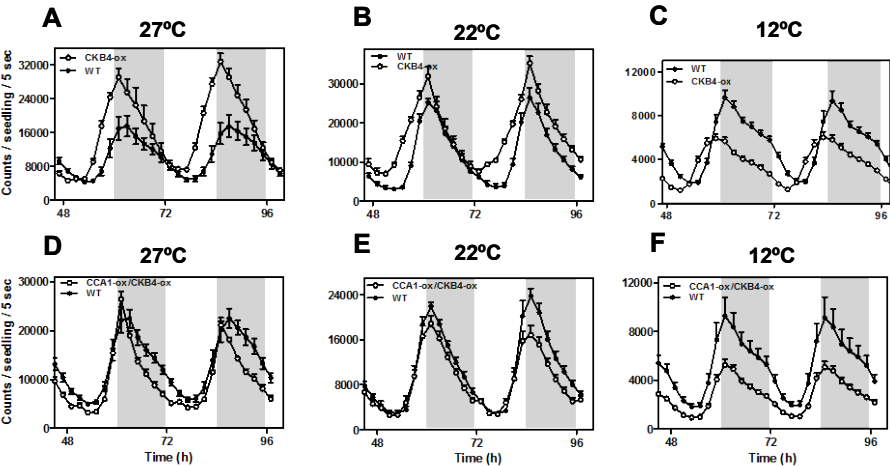

Supplement: Figure S8 — Analysis of TOC1:LUC diurnal expression under different temperatures. Luminescence analysis of TOC1:LUC expression in plants entrained under LD cycles at 22°C and subsequently transferred to LD cycles at 27°C (A, D), 22°C (B, E) or 12°C (C, F). Data is shown as means ± SEM of at least 12 individual seedlings. The experiments were performed at least twice with similar results to those shown here. (0.13 MB PDF) [file pgen.1001201.s008.pdf]

Figure S10

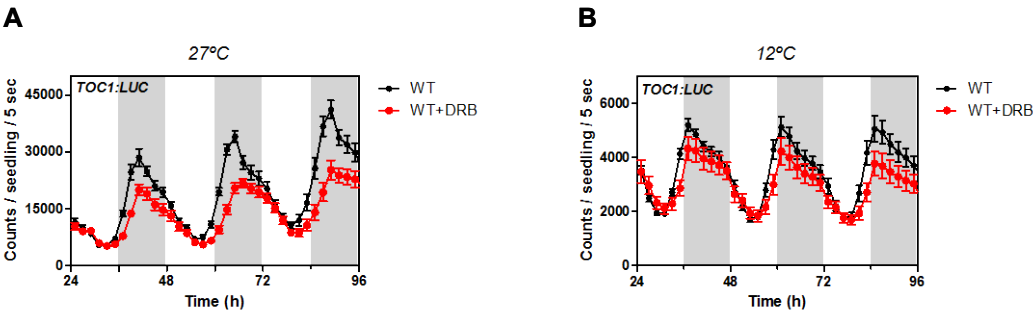

Supplement: Figure S10 — Effects of DRB treatment on TOC1:LUC diurnal expression at different temperatures. TOC1:LUC luminescence in WT plants in the presence or in the absence of 100 µM DRB at 27°C or 12°C. Luminescence was measured in plants entrained under LD conditions at 22°C and transferred to LD cycles at 27°C (A) or 12°C (B). Plots are means ± SEM of 12 individual seedlings. The experiments were repeated at least twice with similar results to those shown here. The white and solid boxes correspond to the light and dark periods, respectively. (0.11 MB PDF) [file pgen.1001201.s010.pdf]

Figure S11

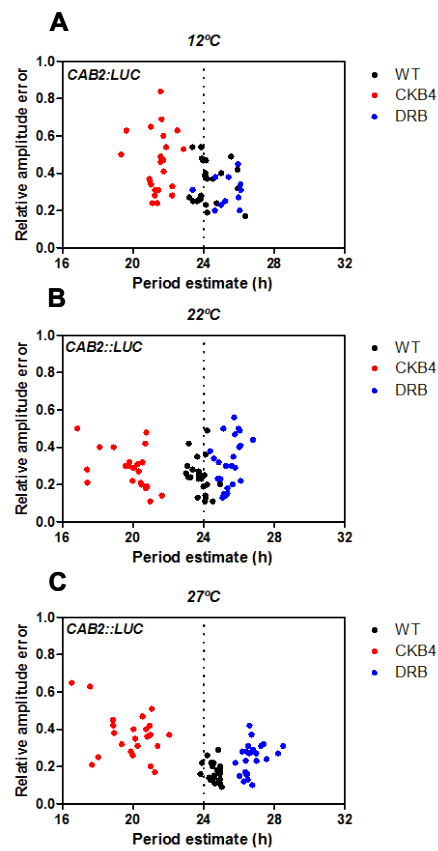

Supplement: Figure S11 — Effects of temperature and CK2 kinase function on the free-running period of the clock-output CAB2:LUC. Free-running periods estimated from CAB2:LUC luminescence signals in WT, CKB4-MYC-ox and WT plants treated with 100 µM DRB at 12°C (A), 22°C (B) and 27°C (C). Period was estimated from individual seedlings plotted against their relative amplitude errors. Data come from two independent experiments with approximately 6-12 plants per phenotype or treatment. (0.12 MB PDF) [file pgen.1001201.s011.pdf]

Figure S12

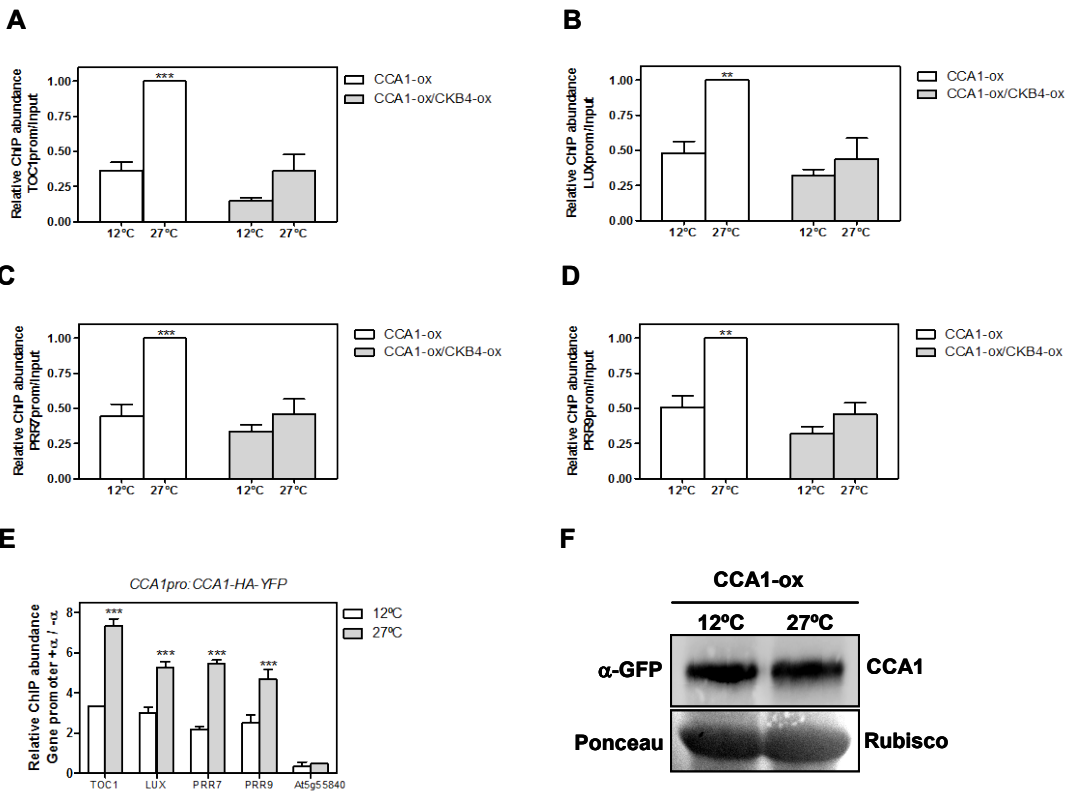

Supplement: Figure S12 — Effects of temperature and CK2 kinase function on CCA1 binding to the promoters of the oscillator genes. Q-PCR analysis of CCA1 binding to TOC1 (A), LUX (B), PRR7 (C) or PRR9 (D) promoters. CCA1-YFP-ox and CCA1-YFP-ox/CKB4-MYC-ox plants were grown under LD cycles at 22°C and transferred to continuous light (LL) at 12°C or 27°C. Samples were collected after 35 h under LL conditions. Data are presented as means ± SD relative to the input and to the maximum value of two independent experiments. (E) Q-PCR analysis of CCA1 binding to TOC1, LUX, PRR7, PRR9 and a clock unrelated gene (At5g55840) in plants expressing CCA1 under its own promoter (CCA1pro:CCA1-HA-YFP). Plants were grown under LD cycles at 22°C and transferred to continuous light (LL) at 12°C or 27°C. Samples were collected after 49.5 h under LL conditions. (F) Western-blot analysis of CCA1 protein accumulation in CCA1-YFP-ox plants at 12°C and 27°C. Similar protein transference in each lane was verified by staining with Red Ponceau. The experiments were performed twice with similar results to those shown here (** p-value<0.01; *** p-value<0.001). (0.17 MB PDF) [file pgen.1001201.s012.pdf]

Figure S13

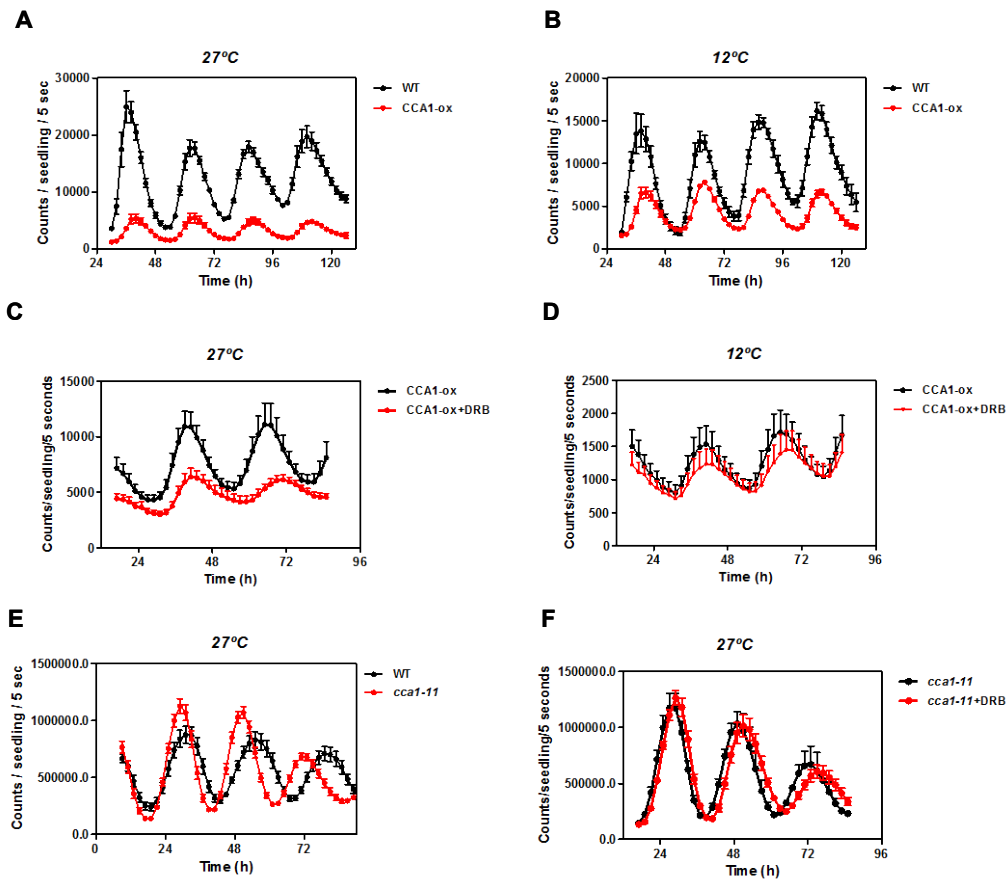

Supplement: Figure S13 — A role for CCA1 regulating circadian gene expression at high temperatures. TOC1:LUC luminescence in WT and CCA1-YFP-ox plants at 27°C (A) or 12°C (B). TOC1:LUC luminescence in WT and CCA1-YFP-ox plants at 27°C (C) or 12°C (D) in the presence or in the absence of 100 µM DRB. (E) CAB2:LUC luminescence in WT and cca1-11 mutant plants at 27°C and in the presence or in the absence of 100 µM DRB (F). Luminescence was measured in plants entrained under LD conditions at 22°C and transferred to LL conditions at the indicated temperatures. Plots are means ± SEM of 12 individual seedlings. The experiments were repeated at least twice with similar results to those shown here. (0.15 MB PDF) [file pgen.1001201.s013.pdf]

Figure S14

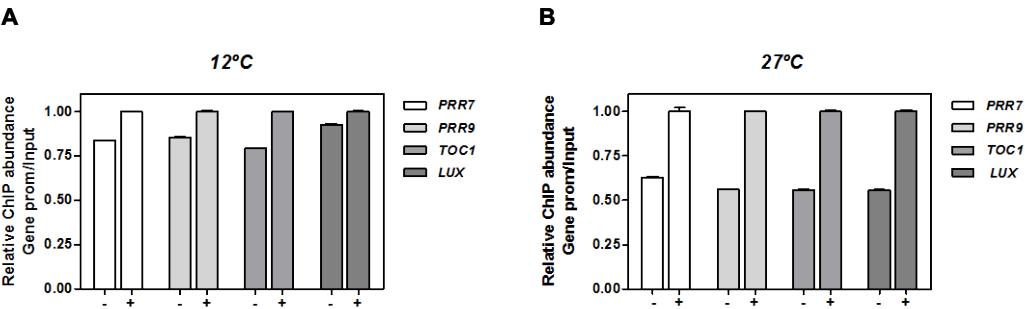

Supplement: Figure S14 — Effects of temperature and CK2 kinase function on CCA1 binding to the promoters of the oscillator genes. Q-PCR analysis of CCA1 binding to TOC1, LUX, PRR7 and PRR9 promoters in plants over-expressing CCA1 in the absence (-) or in the presence (+) of 150 µM of the CK2 inhibitor DRB. Plants were grown under LD cycles at 22°C and transferred to continuous light (LL) at 12°C (A) or 27°C (B). Data are presented as means ± SD relative to the input and to the maximum value. Samples were collected after 50 h under LL conditions. (0.11 MB PDF) [file pgen.1001201.s014.pdf]

Figure S15

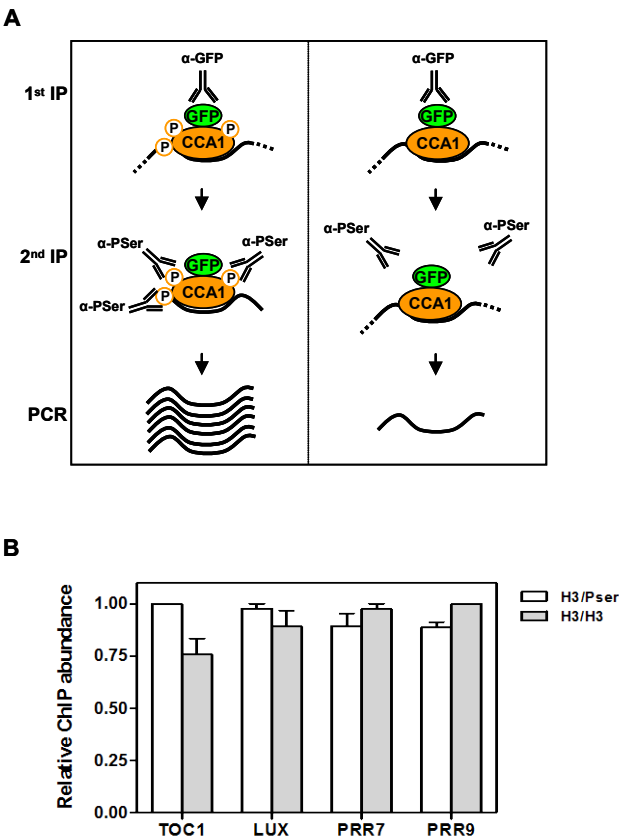

Supplement: Figure S15 — Double ChIP analysis. (A) Schematic representation depicting a summary of the Double-ChIP assay described in Figure 7. A double round of immunoprecipitation (IP) was performed. In the first round, the α-GFP antibody was used to detect total CCA1-YFP protein bound to chromatin. In a second round, the α-PSer antibody was used to specifically detect the phosphorylated isoforms of CCA1 (P). PCR amplification would be obtained only if the phosphorylated CCA1 isoforms are preferentially bound to chromatin. (B) Double-ChIP assays with the combination of α-H3/anti-PSer or α-H3/anti-H3 antibodies Data are presented as means ± SD relative to the maximum value of two independent experiments. (0.13 MB PDF) [file pgen.1001201.s015.pdf]
