# Supplementary material for: The Functional Interplay between Protein Kinase CK2 and CCA1 Transcriptional Activity Is Essential for Clock Temperature Compensation in Arabidopsis
Source: PLoS Genet. 2010 Nov 4;6(11):e1001201. doi: 10.1371/journal.pgen.1001201 (PMC2973838; doi:10.1371/journal.pgen.1001201)
Supplement: Table S1 — Plant Material used in this study. (0.07 MB DOC) [file pgen.1001201.s016.doc]

**Table S1. Plant Material used in this study**

| **REPORTER** | **LINE / ECOTYPE** |
| --- | --- |
| ***TOC1:LUC*** | WT / Col-0 |
|  | CKB4-MYC-ox / Col-0 |
|  | CCA1-ox / Col-0 |
|  | CCA1-YFP-ox / Col-0 |
|  | CCA1-YFP-ox transformed with CKB4-MYC-ox / Col-0 |
|  | CCA1-ox transformed with CKB4-YFP-ox / Col-0 |
|  | CCA1-ox transformed with CKB4-ox / Col-0 |
|  | CKB4-MYC-ox transformed with CCA1-YFP-ox / Col-0 |
|  | CKB4-MYC-ox crossed with CCA1-YFP-ox / Col-0 |
|  | CKA3- / Col-0 |
|  | CKA3- crossed with CCA1-YFP-ox / Col-0 |
|  | cca1-1/lhy-11 / Ler |
|  |  |
| ***CCR2:LUC*** | WT / Col-0 |
|  | CKB4-MYC-ox / Col-0 |
|  | cca1-1/lhyRNAi / Ws-2 |
|  | cca1/lhyR tranformed with CKB4-MYC-ox / Ws-2 |
|  |  |
| ***CAB2:LUC*** | WT / Col-0 |
|  | CKB4-MYC-ox / Col-0 |
|  | CKB4-YFP-ox / Col-0 |
|  | CKB4-cYFP-ox / Col-0 |
|  | CCA1-nYFP-ox / Col-0 |
|  | CKB4-cYFP-ox transformed with CCA1-nYFP-ox / Col-0 |
|  | cca1-11 / Col-0 |
|  | CKB4-cYFP-ox transformed with TOC1-nYFP-ox / Col-0 |
|  | TOC1-YFP-ox / C24 |
|  |  |
| ***NO REPORTER*** | WT / Ler |
|  | WT / Ws-2 |
|  | WT / Col-0 |
|  | CKA3- / Col-0 |
|  | cca1-1/lhy-11/ Ler |
|  | cca1-1/lhy-11 transformed with CKB4-MYC-ox / Ler |
|  | CKB4-MYC-ox / Ler |
|  | CKB4-MYC-ox / Ws-2 |
|  | CCA1-YFP-ox / Col-0 |
|  | CCA1pro::CCA1-MYC / *cca1-1* / Col-0 |
|  | CCA1pro::CCA1-HA-YFP / *cca1-1* / Col-0 |
