# Supplementary material for: The Functional Interplay between Protein Kinase CK2 and CCA1 Transcriptional Activity Is Essential for Clock Temperature Compensation in Arabidopsis
Source: PLoS Genet. 2010 Nov 4;6(11):e1001201. doi: 10.1371/journal.pgen.1001201 (PMC2973838; doi:10.1371/journal.pgen.1001201)
Supplement: Table S2 — Constructs used in this study. (0.05 MB DOC) [file pgen.1001201.s017.doc]

**Table S2. Constructs used in this study**

| **CONSTRUCT** | **REGION** | **VECTOR** |
| --- | --- | --- |
| 35S:CCA1:YFP | CCA1 coding sequence (CDS) , YFP CDS as a tag | pPZP211 |
| 35S:CCA1:nYFP | CCA1 CDS, YFP (nucleotides: 1-462) | pPZP221 |
| 35S:CKB4:MYC | CKB4 CDS, 4x MYC as a tag | pMLBART27 |
| 35S:CKB4:YFP | CKB4 CDS, YFP CDS as a tag | pMLBART27 |
| 35S:CKB4:cYFP | CKB4 CDS, YFP (nucleotides: 463-741) | pMLBART27 |
| TOC1::LUC | Promoter region comprising 1558 bp upstream ATG | pPZPluc |
| 35S:TOC1:YFP | TOC1 CDS, YFP CDS as a tag | pPZP221 |
| 35S:TOC1:nYFP | TOC1 CDS, YFP (nucleotides: 1-462) | pPZP221 |
