# Supplementary material for: The Functional Interplay between Protein Kinase CK2 and CCA1 Transcriptional Activity Is Essential for Clock Temperature Compensation in Arabidopsis
Source: PLoS Genet. 2010 Nov 4;6(11):e1001201. doi: 10.1371/journal.pgen.1001201 (PMC2973838; doi:10.1371/journal.pgen.1001201)
Supplement: Table S3 — Primers used in this study. (0.05 MB DOC) [file pgen.1001201.s018.doc]

**Table S3. Primers used in this study**

| **NAME** | **SEQUENCE** |
| --- | --- |
| TOC1 Prom F | 5'-TTTCTTTCTGTCGACCTTTTG-3' |
| TOC1 Prom R | 5'-AAGGAGATGACGTGGACAATAA-3' |
| LUX Prom F | 5'-CCCGCAAACACAACTTGCT-3' |
| LUX Prom R | 5'-TTCCAACGTGGACGAGTTAGC-3' |
| PRR7 Prom F | 5'-GGTGGCTTATGGTCGTCTCC-3' |
| PRR7 Prom R | 5'-AAGAGTTGAAGAACCACGAATTCTC-3' |
| PRR9 Prom F | 5'-AGGAACAAAAAAAGCCATCCAAT-3' |
| PRR9 Prom R | 5'-GGTATCTTTCGATCACAACG-3' |
| At5g55840 Prom F | 5'-AGAGAGCAACACAGCGAAGGAG-3' |
| At5g55840 Prom R | 5'-CACACTCGAGAGAAACATCCGA-3' |
| PRR9 PrF (Q-PCR) | 5'- AGGACCACCTCCACCGAATC-3' |
| PRR9 PrR (Q-PCR) | 5'- AACAAAGCGGGCCTTCACT-3' |
| PRR7 PrF (Q-PCR) | 5'- CCACTCTCTGGGAGACAGAAAAAC-3' |
| PRR7 PrR (Q-PCR) | 5'- CACAATCAGCTAACGGTTGGG-3' |
| LUX PrF (Q-PCR) | 5'- CCCGCAAACACAACTTGCTA -3' |
| LUX PrR (Q-PCR) | 5'- GGCACGTAAGATGGAGCCA-3' |
| TOC1 PrR (Q-PCR) | 5'-TTTTCTCCAAGATGACGTGGC-3' |
| TOC1 PrF (Q-PCR) | 5'-CCAAGGAGATGACGTGGACAA-3' |
| At5g55840 PrF (Q-PCR) | 5'-AGAGAGCAACACAGCGAAGGAG-3' |
| At5g55840 PrR (Q-PCR) | 5'-CACACTCGAGAGAAACATCCGA-3' |
