# Supplementary material for: The Functional Interplay between Protein Kinase CK2 and CCA1 Transcriptional Activity Is Essential for Clock Temperature Compensation in Arabidopsis
Source: PLoS Genet. 2010 Nov 4;6(11):e1001201. doi: 10.1371/journal.pgen.1001201 (PMC2973838; doi:10.1371/journal.pgen.1001201)
Supplement: Figure S9 — Analysis of TOC1:LUC diurnal expression in CKB4-ox plants under different temperatures. Point-by-point luminescence analysis of TOC1:LUC expression in plants entrained under LD cycles at 22°C and subsequently transferred to LD cycles at 27°C (A) or 12°C (B). Data is shown as means ± SEM of at least 12 individual seedlings. The experiments were performed at least twice with similar results to those shown here. (0.13 MB PDF) [file pgen.1001201.s009.pdf]

Figure S9

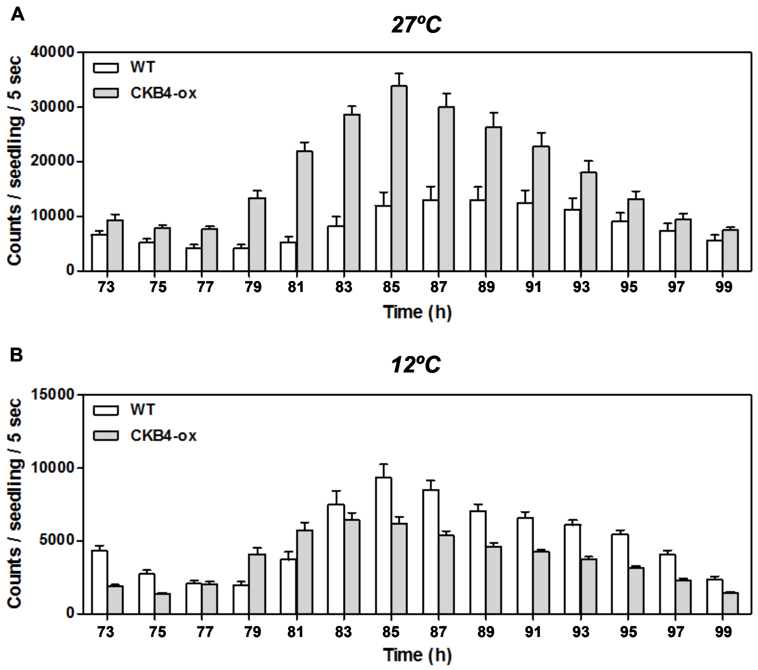

A
27ºC
Counts / seedling / 5 sec
40000
30000
20000
10000
0
WT
CKB4-ox
73
75
77
79
81
83
85
87
89
91
93
95
97
99
Time (h)
B
12ºC
Counts / seedling / 5 sec
15000
10000
5000
0
WT
CKB4-ox
73
75
77
79
81
83
85
87
89
91
93
95
97
99
Time (h)
